# Bullying and depression among adolescents in East Asia: a scoping review on prevalence rates, risk and protective factors

**DOI:** 10.3389/fpsyt.2025.1497866

**Published:** 2025-03-05

**Authors:** Jennifer Sie Hee Kiing, Elizabeth Sarah Ragen, Mohamed Sufyan Bin Mohmed Sulaiman, Wei Sheng Goh, Norman Jun Hao Tan, Sok Hui Ng, Yang Luo, Miny Samuel, Doris Young, Victor Weng Keong Loh

**Affiliations:** ^1^ Department of Paediatrics, Khoo Teck Puat – National University Children’s Medical Institute, National University Hospital, National University Health System, Singapore, Singapore; ^2^ Yong Loo Lin School of Medicine , National University of Singapore, Singapore, Singapore; ^3^ Department of Family Medicine, National University Health System, Singapore, Singapore; ^4^ Division of Family Medicine, Yong Loo Lin School of Medicine, National University of Singapore, Singapore, Singapore

**Keywords:** bullying, depression, adolescent, East Asian, Confucian

## Abstract

**Introduction:**

Bullying and victimization in adolescence is associated with mental health problems including depression. Depression in East Asian adolescents presents similarities and differences from that in Western adolescents. This review reports on the prevalence and psychosocial associations of bullying and depression in East Asian adolescents.

**Methods:**

Electronic databases (Medline, and Embase) were searched for English language articles on bullying and its associations for a span of 10 years (1st January 2013 to 19th January 2024). Searches were limited to studies conducted in East Asia involving adolescents 10-19 years of age.

**Results:**

Out of 1,231 articles initially identified, 65 full-text articles (consisting of 44 cross-sectional and 21 cohort studies) met the inclusion criteria and were included for qualitative synthesis & analysis. Prevalence rates of bullying ranged from 6.1% - 61.3% in traditional bullying victimization and 3.3% to 74.6% in cyberbullying victimization with higher rates in at-risk groups (e.g., adolescents with internet addiction). Psychosocial associations of bullying and depression which were similarly found in Western cultures include individual factors of coping style and gender; family factors of functioning and sibling relationships; and community factors of friendship and school-connectedness. In contrast, unique East Asian risk factors included being different (i.e., sexual minority status) and teachers as bullies.

**Conclusion:**

Findings of this scoping review suggest that strong relationships within families, peers and the school community coupled with adolescents’ positive coping style are protective against the negative effects of bullying. Conversely, poor parent-child attachment in the midst of family dysfunction, poor engagement with peers and the school community together with low self-esteem predispose East Asian adolescents to depressive symptoms as a result of victimization. Similar to Western cultures, adolescents who are bully-victims and poly-victims are most vulnerable to depression. As a significant proportion of bullying occurred in school, future research could focus on a whole-school intervention approach to counter bullying.

## 1 Introduction

Depression is estimated by the World Health Organization (WHO) to occur in up to 2.8% of adolescents (1). Evidence suggests that bullying and victimization during early adolescence is associated with depression and suicidality during late adolescence, which may persist into adulthood (2, 3).

Bullying is understood to be one key risk factor for depression (4–7). Globally, about a third of adolescents aged 12 – 15 years have experienced bullying with suicide attempts twice as likely in adolescents who have experienced bullying compared to those who have not (8). While studies published in Western countries have shown a link between bullying, depression and suicidality in adolescents (5–7, 9), data from East Asian countries is sparse. Studies have shown many similarities in bullying patterns between Western and Eastern cultures (4, 8), but there is limited information on differences. The culture of East Asian countries does appear to impact the experience of school bullying especially in how it is experienced, prevented or mitigated (10). For instance, while students in England reported bullying in the playground from older and unfamiliar school mates, students in Japan and Korea reported bullying from classmates they knew well (8). A better understanding of the differences in bullying patterns can serve to inform practices to prevent and address bullying in East Asian cultures.

In East Asian countries, ‘collectivism’ which has its roots in Confucianism, provides the basis for how society functions. ‘Collectivism’ refers to a culture where the goals of the group are prioritized over the goals of individuals for the sake of harmony. Elements of collectivism include harmonious interpersonal relationships, group orientation, hierarchy, compliance with authority, and avoidance of peer and interpersonal conflicts” (11, 12). With its emphasis on hierarchy, power differences can be marked between adults in positions of authority and adolescents in their care. Adolescents may have little recourse when victimization originates from persons in authority (9). While East Asian culture promotes helping the vulnerable and weak within a group (13), the goals of maintaining harmony within a group may also mean that individuals who do not conform to the rules of the group may be exposed to correction by others within the group (14). The culture of East Asian countries does impact the way bullying is experienced or mitigated from differences in societal values or school systems (15). For instance, school bullying in collectivistic cultures may more likely be in the form of group bullying by social isolation compared to aggression in individualistic cultures (16) and more bullying may occur in hierarchical classrooms in Eastern cultures compared to more consultative classrooms in western cultures (15).

The emphasis on group goals in East Asian cultures during adolescence offers a contrast with personal goals of adolescence in Western cultures to achieve self-identity (Who am I)? and autonomy (Do my choices matter)? (17). Would the hierarchical norms and focus on harmonious interpersonal relationships mean that East Asian adolescents have fewer opportunities to receive support in the face of victimization, and stop bullying?

The aims of this review were to understand if there were unique aspects of East Asian, ‘collectivistic’, culture which would predispose adolescents to bullying and depression or protect them when bullying occurred. Countries/regions where the mainstream culture is Confucian-influenced (18) were selected for this study specifically including China, Japan, South Korea, Macau, Mongolia, Taiwan, Hong Kong and Singapore. The prevalence of bullying among adolescents in the cultures of East Asia and both risk and protective factors for bullying and development of depression in East Asian adolescents were investigated.

## 2 Methods

This review was conducted in line with the “Preferred Reporting Items for Systematic Reviews and Meta-Analyses extension for Scoping Reviews” (PRISMA-SCR) standardized reporting guidelines (see **Appendix** for checklist).

### 2.1 Information source and search strategy

A systematic search was conducted in 2 databases, MEDLINE (using the PubMed platform) and Embase for English language articles published from 1st January 2013 to 19th January 2024. These two databases were prioritized to yield a good scope of articles for the topic of interest. Searches were limited to studies conducted in populations from these countries: Japan, South Korea, Mongolia, China, Hong Kong, Macau, Taiwan, Singapore.

To achieve the maximum sensitivity of the search strategy, we used combinations of free text and medical subject heading (MeSH) terms. The key concepts used for this search included “adolescent” (population) AND “bullying” (exposure) AND “depression” (outcome). Each concept was expanded, including the use of MeSH terms, with each term for the same concept searched using the Boolean operator “OR”, while the 3 main concepts combined with the Boolean operator “AND” for the overall search. The reference lists of included studies were also screened to identify other relevant studies.

### 2.2 Inclusion criteria

Studies which met the following inclusion criteria were included: (i) involved adolescents 10 – 19 years old (19) from East Asian countries, specifcially including China, HK, Mongolia, Macau, South Korea, Japan, Taiwan and Singapore; (ii) examined traditional and/or cyberbullying (see **Supplementary Table 1** for definitions) as the exposure; (iii) had psychosocial associations of bullying and development of depression as the outcome (Bullying was defined by its modalities - direct i.e., fighting and aggression versus indirect i.e., spreading rumours; types i.e., physical, relational, verbal; and forms i.e., traditional vs cyberbullying i.e., internet or social media based (20); single type versus poly-victimization involving multiple forms of bullying (see **Supplementary Table 1** for definitions); and (iv) contained original epidemiological research i.e., cross-sectional and cohort studies.

### 2.3 Exclusion criteria

Studies which (i) involved East Asian expatriate adolescents; (ii) did not have outcomes related to bullying or bullying as a form of exposure; (iii) did not measure depression with standardized or validated scales; (iv) were conducted in South East Asian countries including Brunei, Malaysia, Indonesia, Vietnam, Cambodia, Philippines, Laos, Myanmar, Thailand, and Timor Leste where Confucianism is not practised widely (Singapore, which adopts a Confucian-informed culture, was not excluded); (v) were informal publications (such as commentaries, letters to the editor, editorials, meeting abstracts, theses or dissertations); (vi) were not published in English; (vii) lacked access to full texts; or (viii) were review papers, were excluded.

### 2.4 Study selection

Articles retrieved through the searches of the 2 databases were screened to remove duplicates. Four authors (G.W.S., N.T.J.H., N.S.H., L.Y.) independently assessed the abstracts and full-texts of articles identified from the searches to ensure they met the study inclusion and exclusion criteria described above. Any disagreements were resolved by discussion and consensus with the lead author (J.S.H.K.).

### 2.5 Data extraction

Four authors (M.S.B.M.S., G.W.S., N.T.J.H., N.S.H., L.Y.) extracted relevant data using a standard template including study author, country, study design, settings (e.g., urban or rural), sample size, participant characteristics (e.g., age), description of exposures and definitions, adjusted factors, outcomes (e.g., depression) and study design limitations.

### 2.6 Quality assessment

The risk of bias from cohort and cross-sectional studies included in this review were evaluated using the Newcastle-Ottawa Quality Assessment (NOS) Scale which assesses the quality of non-randomized studies (21). High quality studies with a low risk of bias are scored between 7 and 9; fair quality studies with moderate-high risk of bias and low-quality studies with very high risk of bias are given scores between 4 and 6 and 0 and 3, respectively.

### 2.7 Synthesis of results

As this was a scoping review, a qualitative synthesis approach was adopted. More emphasis was placed on cohort and cross-sectional studies of “good” quality and low risk of bias (described as NOS score ≥ 7) with larger sample sizes (n > 1,000). P-values were represented as * where *p* < 0.001 was ***, *p* < 0.01 was ** and *p* < 0.05 was*.

## 3 Results

**Figure 1:**
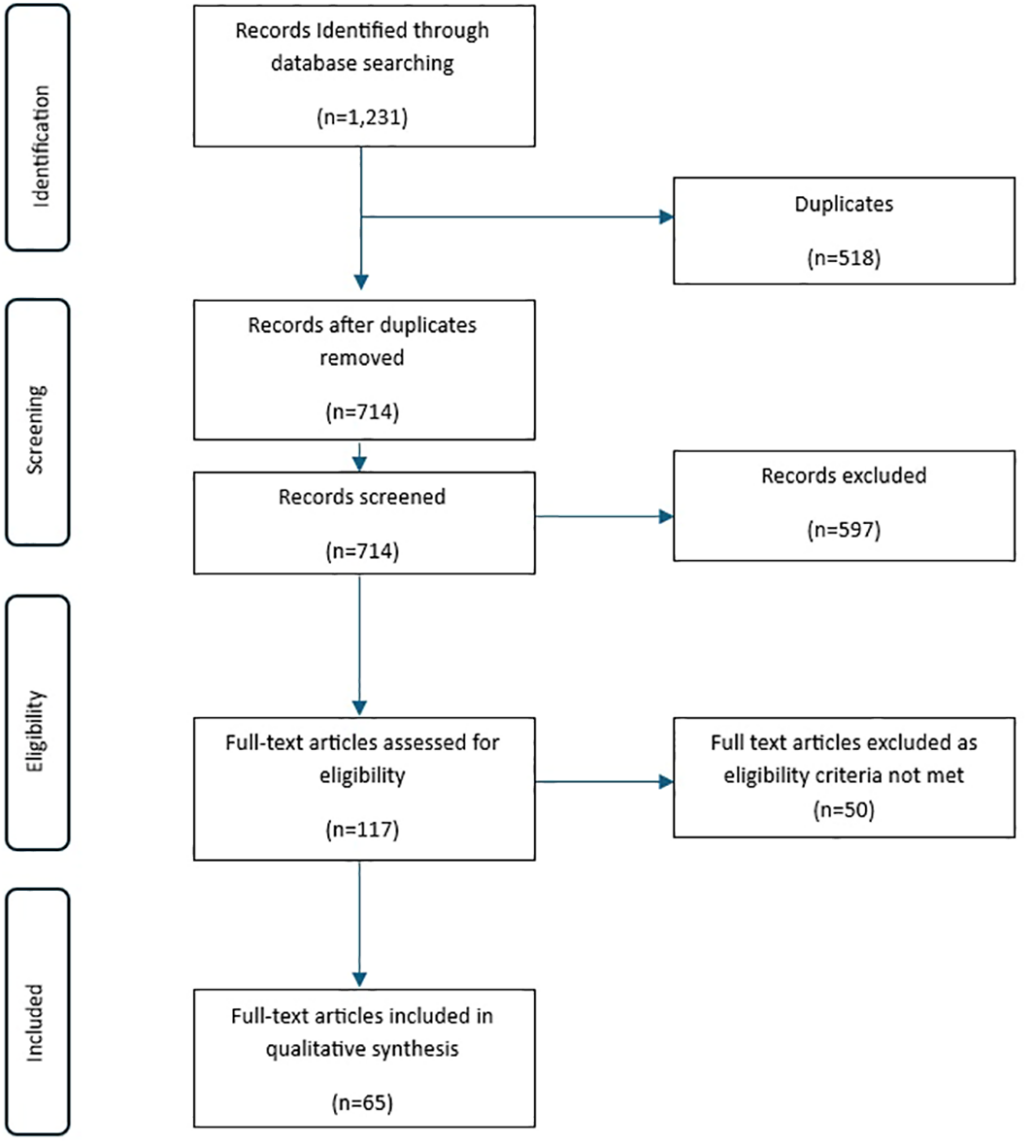
PRISMA flow diagram illustrating search process and records screening.

A total of 1,231 articles were identified from Medline (n = 764) and Embase (n = 467) databases. After removal of 518 duplicates, 714 records were eligible for further screening. Of these, 597 studies did not meet the eligibility criteria, leaving 117 studies for further evaluation. Of these, 51 were excluded for reasons such as unavailability of full-text and non-East Asian study population. Thus, a total of 65 studies were included in this review for analysis, comprising 21 cohort (22–42) studies [Taiwan (n = 3), South Korea (n = 2), China (n = 16), and 44 cross-sectional (43–86) studies [China (n = 27), HK (n = 1) Taiwan (n = 9), Korea (n = 6), and Japan (n = 1)]. The study screening and selection process is shown in **Figure 1**, while a detailed description of the included studies is provided in **Table 1**.

**Table 1:** Characteristics of included studies (n = 66).

Author (year); Country [Ref]	Study design (Sample size; age)	Study Setting; Study period	Measurement of bullying and Definition	Measurement of depression	Outcomes of interest
Cohort studies (n = 21)					
Kawabata et al. (2014); Taiwan (22)	n=387 (mean age 10.48 years)	Urban; over 6 months in unspecified time period	Unnamed questionnaire on victimization Self-report questionnaire (victim)	Teacher Report Form	Depression
Chang et al. (2019); Taiwan (23)	n=4072 (mean age 16.18 years for boys; mean age 16.08 years for girls)	Urban; 2009-2011	Unnamed questionnaire on victimization	Center for Epidemiological Studies Depression (CES-D); Children’s Depression Inventory (CDI)	Depression
Hong et al. (2018); South Korea (24)	n=1750 (age range: 13-19 years)	Urban; 2011-2016	Korean Youth Panel Survey Self-report questionnaire (victim)	Korean Children and Youth Panel Survey (KCYPS)	Depression
Chu et al. (2019); China (25)	n=661 (mean age=12.86 years)	Urban; 2016-2017	Traditional Bullying Scale; Chinese version of the Revised Cyberbullying Inventory Self-report questionnaires on traditional and cyberbullying (victim)	Chinese version of the 21-item Depression Anxiety Stress Scale (DASS–21)	Depression, general anxiety, stress, low self-esteem, social anxiety, and loneliness
Chang et al. (2017); Taiwan (26)	n=1893 (mean age=14.66 years)	Urban and rural; 2009-2012	Social Experiences Questionnaire Self-report questionnaire (victim)	CES-D	Depression
Chen & Chen (2020); China (27)	n=1507 (age range: 12-15 years)	Urban; over 1 year in unspecified period	Unnamed questionnaire on victimization nominated by peers Peer report questionnaire (victim)	CDI	Depression
He et al. (2022); China (28)	n=1687 (mean age=12.49 years)	Urban; 2019-2020	Self-report questionnaire on school bullying adopted/modified from previous studies 8-item Self-report questionnaire on bullying victimization and perpetration	Centre for Epidemiological Studies Depression Scale for Children (CES-DC) (Chinese Version)	Depression, sleep problems
Yang et al. (2022); China (29)	n=450 (mean age=11.63 years)	Urban; over 3 years in unspecified period	Self-report measure adapted from OBVQ (Chinese)	CES-DC	Depression
Xiong et al. (2023); China (30)	n=2551 (mean age=12.99 ± 0.61 years)	Urban; Jan-June 2015	OBVQ (Chinese)	CDI	Depression
Li et al. (2023); China (31)	n=10,297, 7^th^ graders	Urban & rural; 2 and 5 year follow up from 2013-2021	Slightly modified California Bullying Victimization Scale	CES-D	Depression; worse healthy lifestyles; poorer sleep quality lower academic achievement
Yuan et al. (2021); China (32)	n=1390 (mean age =15.19 years)	Urban/Rural (unspecified) over 6 months (unspecified	Revised Cyber Bullying Inventory-cyberbullying Subscale 9RCBPI-CA) - Chinese	CES-D (Chinese)	Depression
Ren et al. (2023); China (33)	n=1911 (mean age=12.98 ± 0.60 years) at T1	Urban, Jan 2015 – Jun 2016	Chinese Peeer Victimzation Scale for Children and Adolescents (CPVSCA); adapted fron OBVQ	CDI	Depression
Yan et al. (2023); China (34)	n=592 (mean age at T1 = 9.43 years (females); 9.56 years (males)) Age range: 8-12 years	2 year (2018-2020)	OBVQ (Chinese)	Children Depression Scale-Depression Scale for Children (CDS-DC)	Depression, Non-Suicidal Self Injury, Self-compassion, Negative thoughts
Gao et al. (2021); China (35)	n=2407 (male =1191; mean age=12.75 ± 0.58 years)	12 months	Cyber victimization Scale (Edur and Kavsut) Chinese version	CESD Chinese	Depressive symptoms, peer pressure,
Perret et al. (2021); South Korea (36)	n=2258; (mean age=15.7 years)	Urban and rural; 3 years 2011-2013	Korean School Violence Victimization Questionnaire	Symptom Checklist-90 Revised (SCL-90 revised)- depression subscale	Depression
Yang et al. (2023); China (37)	n=2339 (mean age=12.97 ± 0.58 years)	Urban; December 2015-June 2016	OBVQ (Chinese)	CDI	Depression
Liang et al. (2023); China (38)	n=3510 (mean age=12.36 years)	Urban; over 1 year in unspecified period	OBVQ (Chinese)	CDI- short version (CDI-S)	Depression, non-suicidal self-injury
Shen et al. (2023); China (39)	n=1205 (mean age=11.27 years) Age range: 9-15 years	Urban or rural (insufficient info); 2017-2019	Multidimensional Peer Victimization Scale	Center for Epidemiologic Studies Depressive Symptoms Scale (CES-DS)	Depression
Long, Zhou & Li (2020); China (40)	n=447 (7^th^ and 8^th^ graders)	Urban; over 6 months, unspecified period	Peer nomination relational victimization items from Children’s Social Experience Questionnaire (Crick and Bigbee 1998)	CDI-S (Children’s depression inventory short form) SASC-R (social anxiety scale for children revised)	Depression, Anxiety
Yu et al. (2023); China (41)	n=1711 (mean age=11.9 ± 1.6 years)	Urban and rural; 2017	OBVQ (Chinese)	CES-D	Depression
Zhao & Li (2022); China (42)	n=691 (mean age=12.74± 0.43 years)	Urban and rural; over two years, unspecified time period	OBVQ (Chinese)	CES-D	Depression
Cross Sectional Studies (n=44)					
Chan (2013); China (43)	n=18341 (mean age=15.9 years)	Urban and rural; 2009-2010	Juvenile Victimization Questionnaire (JVQ) [Self-report questionnaire (victim)]	Beck Depression Inventory II (Chinese version)	Depression, health-related quality of life, posttraumatic stress disorder, deliberate self-harm, and suicide ideation
Chang et al. (2015); Taiwan (44)	n=1867 (age range: 13-15 years)	Urban and rural; 2013	Four questions on cyberbullying victimization [Self-report questionnaire of cyber bullying (victim, bully) and internet use]	Center for Epidemiologic Studies Depression Scale (CES-D)	Depression
Chang et al. (2013); Taiwan (45)	n=2992 (age range: 15-16 years)	Urban; 2010	Questionnaire developed based on the US Youth Risk Behavior Surveillance System & Youth Internet Safety Survey [Self-report Questionnaire (victim, bully)	CES-D	Depression
Chen et al. (2020); Taiwan (46)	n=2419 (age range: 13-18 years)	Urban; 2009	California School Climate and Safety Survey (CSCSS) [Self-report questionnaire (victim)]	Brief Symptom Rating Scale (BSRS)	Depression
Chen et al. (2018); China (47)	n=18341 (mean age=15.86 years)	Urban; 2009-2010	Peer and sibling module of the of Juvenile Victimization Questionnaire (JVQ) Relational Aggression Scale (RAS) [Self-report questionnaire (victim, bully)]	Beck Depression Inventory II	Depression, PTSD, health-related quality of life, deliberate self-harm and suicide ideation
Chen et al. (2018); Hong Kong (48)	n=2120 (mean age=15.11 years)	Urban; unspecified time period	Three questions about doxing experience Self-report Questionnaire of cyberbullying (victim)	Depression Anxiety Stress Scale, 21-item (DASS-21)	Depression, anxiety, stress
Chu et al. (2018); China (49)	n=489 (mean age=12.67 years)	Urban; unspecified time period	Cyberbullying Inventory (CBI) Self-report Questionnaire of cyberbullying (victim)	CES-D	Depression, anxiety
Guo et al. (2020); China (50)	n=1252 (age range: 15-18 years)	Rural; 2017	National Center for Education Statistics’ School Survey on Crime and Safety [Self-report questionnaire (victim)]	CES-D	Depression
He et al. (2019); China (51)	n=6576 (mean age=13.37 years)	Urban; 2015	Questions about conflict with peers, teachers and school connectedness Teacher and peer relations questionnaire	Depression Self-Rating Scale for Children (DSRSC)	Depression
Hong et al. (2018); South Korea (52)	n=10453 (mean age=15 years)	Urban; 2015	Korean National Youth Policy Institute, Cyberbullying Experiences Questionnaire (CEQ) Self-report Questionnaire of cyberbullying (victim)	Youth Self-Report, Korean version (K-YSR)	Depression
Hong et al. (2016); China (53)	n=20511 (mean age=16.3 years)	Urban; 2011-2012	Questions about bullying [Self-report questionnaire (victim, bully)]	CES-D	Depression, suicidal ideation
Hu et al. (2016); Taiwan (54)	n=287 (mean age=13.1 years)	Urban; 2012-2013	School Bullying Experience Questionnaire (Chinese Version) [Self-report questionnaire (bully)] plus ADHD/ASD reports	CES-D	Depression, anxiety
Jung et al. (2014); South Korea (55)	n=4531 (age range: 11-14 years)	Urban; unspecified time period	Cyberbullying and Online Aggression Survey [Self-report questionnaire on cyberbullying and internet use (victim, bully)]	Youth Self-Report, Korean version (K-YSR)	Depression
Kozasa et al. (2017); Japan (56)	n=827 (mean age=11.26 years for pre-adolescents; mean age=13.76 years for adolescents)	Urban; 2014	Questions about bullying in school Survey on 6 types of bullying (victim, bully)	Youth Self Report (YSR)	Depression, suicidal ideation
Lee & Kim (2017); Korea (57)	n=2283 (mean age=14 years)	Urban and rural; unspecified time period	Questions about victimization n bullying [Self-report survey (victim, bully)]	Unvalidated: four questions	Depression
Li et al. (2018); China (58)	n=1742 (age range: 16-17 years)	Urban and rural; 2016	Centers for Disease Control and Prevention’s (CDC) Youth Risk Behavior Survey (YRBS) Self-report questions (victim)	Centers for Disease Control and Prevention’s (CDC) Youth Risk Behavior Survey (YRBS)	Depression
Ma et al. (2018); Taiwan (59)	n=730 (mean age: 12.8 years)	Urban; unspecified time period	Modified Peer Victimization Scale Self-report questionnaire (victim)	CDI	Depression, loneliness
Min et al. (2015); South Korea (60)	n=1198 (age range: 12-13 years)	Urban; 2012	Olweus Bully/Bully-Victim Questionnaire (BVQ) Self-report questionnaire (bully, victim)	CES-D	Depression, suicidal ideation
Seo et al. (2017); South Korea (61)	n=2936 (mean age=13.8 years)	Urban; 2014	Unnamed questionnaire about victimization Self-report questionnaire (victim)	CDI	Depression
Shao et al. (2014); China (62)	n=2457 (mean age=12.6 years)	Urban; 2009	Olweus Bully/Bully-Victim Questionnaire (BVQ) Self-report questionnaire (bully, victim)	CDI	Depression, loneliness, anxiety, academic achievement
Tang et al. (2018); China (63)	n=1663 (mean age=13.9 years)	Rural; 2017	Olweus Bully/Bully-Victim Questionnaire (BVQ) Self-report questionnaire (bully, victim)	Kutcher Adolescent Depression Scale (KADS)	Depression, panic, psychological distress, self-esteem,
Wang et al. (2019); China (64)	n=1347 (median age=12.5 years)	Rural; 2017	Yes/no question based on Olweus Self-report questionnaire (victim)	Patient Health Questionnaire (PHQ-9)	Depression, anxiety, suicidal ideation
Wang et al. (2020); China (65)	n=569 (mean age=11.75 years)	Urban; unspecified time period	Olweus Bully/Bully-Victim Questionnaire (BVQ) Self-report questionnaire (bully, victim)	CDI	Depression, loneliness
Xiong et al. (2019); China (66)	n=194 (mean age=13.51 years)	Rural; 2017	Multidimensional Peer-Victimization Scale Self-report questionnaire (victim)	CES-D	Depression, loneliness, self-harm
Yeh et al. (2019); Taiwan (67)	n=474 (mean age=11.0 years)	Urban; 2009-2012	Chinese version of the School Bullying Experience Questionnaire (C-SBEQ) Self-report questionnaire (bully, victim)	CDI	Depression, pain, pain-induced functional impairment, anxiety, sleep quality
Yen et al. (2014a); Taiwan (68)	n=6406 (mean age=14.8 years)	Urban and rural; 2009	Chinese version of the School Bullying Experience Questionnaire (C-SBEQ) Self-report questionnaire (bully, victim)	CES-D	Depression, anxiety, insomnia, social phobia, ADHD, suicidal ideation, alcohol abuse
Yen et al. (2014b); Taiwan (69)	n=5252 (mean age=14.9 years)	Urban and rural; 2009	Chinese version of the School Bullying Experience Questionnaire (C-SBEQ) Self-report questionnaire (bully)	CES-D	Depression, social phobia, suicidal ideation, self-esteem
Yen et al. (2014c); Taiwan (70)	n=251 (mean age=13.1 years)	Urban; 2012-2013	School Bullying Experience Questionnaire (C-SBEQ) (Chinese version); Korean National Youth Policy Institute, Cyberbullying Experiences Questionnaire (CEQ) Self-report questionnaire on cyberbullying (bully)	CES-D	Depression, anxiety, suicidal ideation
Yin et al. (2017); China (71)	n=755 (mean age=13.52 years)	Urban; unspecified time period	Olweus Bully/Bully-Victim Questionnaire (BVQ) Self-report questionnaire (victim)	CES-D	Depression
Yun & Kim (2016); South Korea (72)	n=1793 (mean age=13.9 years)	Urban; 2013	Olweus Bully/Bully-Victim Questionnaire (BVQ) Survey of 5 types of bullying behaviour (bully)	Korean version of the Minnesota Multiphasic Personality Inventory-Adolescent (MMPI-A)	Depression, anxiety, self-harm, suicidal ideation,
Zhou et al. (2017); China (73)	n=448 (mean age=10.82 years)	Urban; unspecified time period	One item from Olweus Bully/Bully-Victim Questionnaire (BVQ) Self-report questionnaire (victim)	CES-D	Depression
Pan & Spittal (2013); China (74)	n=8182 (age range: 12-18) years	Urban; 2003	Unnamed questionnaire on victimization Self-report questionnaire (victim)	Unvalidated question about depressive symptom	Depression, anxiety, suicidal ideation fighting, injury inflicted, smoking and alcohol abuse
Zhao et al. (2021); China (75)	n=16,380 (mean age ~ 15 years) Age range: 11-20 years	Urban & rural; 2018	Olweus Bullying Questionnaire (OBQ)	CES-D (Chinese)	Depression, Anxiety, Suicidal Ideation, Attempt, NSSI (non-suicidal self-injury)
Mei et al. (2021); China (76)	n=2956 (mean age=13.39 ± 1.03 years)	Urban; Dec 2017-Jan 2018	Olweus Bully/Victim Q (OBVQ) Chinese	CES-D (Chinese)	Social anxiety, Depression, Sleep duration
Lai et al. (2023); China (77)	n=19,809	Urban & rural; 2019 SCAHS	Olweus Bully/Victim Q (OBVQ) Chinese	CES-D (Chinese)	Anxiety, depression
Liu et al. (2020); China (78)	n=8918 (mean age=14.55 ± 1.63 years)	Urban & rural; 2018	Olweus Bully/Victim Q (OBVQ) Chinese	GAD-7	Anxiety, depression
Guo et al. (2022); China (79)	n=3635 (junior 1 to senior 2)	Rural; unspecified time period	Olweus Bully/Victim Questionnaire	9-item Patient Health Questionnaire (PHQ-9) (Chinese version)	Depression, anxiety, aggressive behaviour, subjective wellbeing
Peng et al. (2022); China (80)	n=3062; Age range: 14-18 years	Urban & rural Apr – July 2018	OBVQ (Chinese)	PHQ-9 (chinese)	Depression, anxiety, sibling bullying victimization
Zhu et al. (2020); China (81)	n=18,452; Age range: 15-17 years	Urban; 2009 & 2010	Chinese Juvenile Victimization Questionnaire (JVQ Cahn et al., 2011)	Beck Depression Inventory Version II	Self-esteem, Depression, Health status
Wen et al; China (2022) (82)	n=1481 (mean age=16.67 years) Age range: 14-19 years	Urban (1 large regional high school)	Illinois Bully Scale	GAD- 7 Chinese,	Anxiety, Depression
Zhu et al. (2021); China (83)	n=3232 (age range: 15-17 years)	Urban and rural; September 2009-June 2010	Relational Aggression Scale (modified)	Beck Depression Inventory II	Depression, PTSD, problem drinking, cigarette smoking, gambling engagement
Cao et al. (2021); China (84)	n=2022 (mean age=13.4 ± 1.0 years) Age range: 10-17 years	Urban; unspecified period	Olweus Bully/Victim Q (OBVQ) Chinese (Solberg & Olweus 2003)	CES-D	Internet addiction, sleep quality, Depression
Liu et al. (2023); China (85)	n=3841 (grade 1 and 2 of senior high school)	Urban and rural; April-July 2018	OBVQ (Chinese)	PHQ-9 (Chinese) GAD-7 (Chinese)	Depression, anxiety
Fan et al. (2021); China (86)	n=1174 (mean age=15.45 ± 2.25 years)	Urban and rural; time period unspecified	OBVQ (Chinese)	CES-D	Depression

We assessed the quality of the included studies using the NOS. Findings are summarized in **Tables 2**, **3**. Of the 44 cross-sectional studies with an average NOS score of 7.47 (range: 4 – 9); 35 had a low risk of bias (NOS score ≥ 7), 9 had an intermediate risk of bias (NOS score 4 – 6); with no studies having a high risk of bias (NOS score 0 – 3). Of the 21 cohort studies with an average NOS score of 6.28 (range: 3 – 8); 13 had a low risk of bias (NOS score ≥ 7), 7 had an intermediate risk of bias (NOS score 4 – 6) and 1 had a high risk of bias (NOS score 0 – 3).

**Table 2:** Quality Assessment for cross-sectional studies (n=45) using the Newcastle-Ottawa Scale adapted for cross-sectional studies.

Author (Year); Country [Ref]	Selection Criteria				Comparability	Outcome		Total
	*Representativeness of the sample*	*Sample size*	*Non-respondents*	*Ascertainment of the exposure (risk factor)*	*The subjects in different outcome groups are comparable, based on the study design or analysis. Confounding factors are controlled.*	*Assessment of the outcome*	*Statistical test*	
Chan (2013); China (43)	1	0	1	2	2	1	1	8 Good
Chang et al. (2015); Taiwan (44)	1	0	0	2	2	1	1	7 Good
Chang et al. (2013); Taiwan (45)	1	0	0	2	2	1	1	7 Good
Chen et al. (2020); Taiwan (46)	1	0	0	2	2	1	1	7 Good
Chen et al. (2018); China (47)	1	0	0	2	2	1	1	7 Good
Chen et al. (2018); Hong Kong (48)	1	0	0	2	0	1	1	5 Fair
Chu et al. (2018); China (49)	0	0	0	2	2	1	1	6 Fair
Guo et al. (2020); China (50)	1	1	0	2	2	1	1	8 Good
He et al. (2019); China (51)	1	0	0	2	2	1	1	7 Good
Hong et al. (2018); South Korea (52)	1	0	0	2	2	1	1	7 Good
Hong et al. (2016); China (53)	1	0	0	1	2	1	1	6 Fair
Hu et al. (2016); Taiwan (54)	0	1	1	2	2	1	0	7 Good
Jung et al. (2014); South Korea (55)	1	1	0	2	1	1	1	7 Good
Kozasa et al. (2017); Japan (56)	1	1	0	1	1	1	1	6 Fair
Lee & Kim (2017); Korea (57)	1	1	0	1	1	1	1	6 Fair
Li et al. (2018); China (58)	1	1	0	1	2	1	1	7 Good
Ma et al. (2018); Taiwan (59)	1	1	0	2	1	1	1	7 Good
Min et al. (2015); South Korea (60)	1	1	0	2	0	1	0	5 Fair
Seo et al. (2017); South Korea (61)	1	1	0	1	0	1	1	5 Fair
Shao et al. (2014); China (62)	1	1	0	2	2	1	0	7 Good
Tang et al. (2018); China (63)	1	1	1	2	2	1	1	9 Good
Wang et al. (2019); China (64)	1	1	0	1	2	1	1	7 Good
Wang et al. (2020); China (65)	1	1	0	2	1	1	1	7 Good
Xiong et al. (2019); China (66)	1	1	0	2	1	1	1	7 Good
Yeh et al. (2019); Taiwan (67)	1	1	1	2	2	1	1	9 Good
Yen et al. (2014a); Taiwan (68)	1	1	0	2	2	1	1	8 Good
Yen et al. (2014b); Taiwan (69)	1	1	1	2	2	1	1	9 Good
Yen et al. (2014c); Taiwan (70)	1	1	0	1	2	1	1	7 Good
Yin et al. (2017); China (71)	1	1	0	2	2	1	1	8 Good
Yun & Kim (2016); South Korea (72)	1	1	1	2	2	1	1	9 Good
Zhou et al. (2017); China (73)	1	1	0	2	2	1	0	7 Good
Pan & Spittal (2013); China (74)	1	1	1	2	2	1	1	9 Good
Zhao et al. (2021); China (75)	1	1	1	1	2	1	1	8 Good
Mei et al. (2021); China (76)	1	1	1	2	2	1	1	9 Good
Lai et al. (2023); China (77)	1	1	1	2	2	1	1	9 Good
Liu et al. (2020); China (78)	1	0	1	1	2	1	1	7 Good
Guo et al. (2022); China (79)	1	0	0	2	0	1	1	5 Fair
Peng et al. (2022); China (80)	1	0	1	2	2	1	1	8 Good
Zhu et al. (2020); China (81)	1	0	1	1	2	1	1	7 Good
Wen et al; China (2022) (82)	0	0	0	1	2	1	1	4 Fair
Zhu et al. (2021); China (83)	1	1	1	1	2	1	1	8 Good
Cao et al. (2021); China (84)	1	1	1	2	2	1	1	9 Good
Liu et al. (2023); China (85)	1	1	1	2	2	1	1	9 Good
Fan et al. (2021); China (86)	1	1	1	2	2	1	1	9 Good

**Table 3:** Quality Assessment for cohort studies (n=21) using the Newcastle-Ottawa Scale.

Author (Year); Country [Ref]	Selection Criteria				Comparability	Outcome			Total
	*Representativeness of the sample*	*Selection of the non-exposed cohort*	*Ascertainment of the exposure*	*Demonstration that outcome of interest was not present at start of study*	*Comparability of cohorts on the basis of the design or analysis controlled for confounders*	*Assessment of the outcome*	*Was follow-up long enough for outcomes to occur*	*Adequacy of follow-up of cohorts*	
Kawabata et al. (2014); Taiwan (22)	1	1	0	0	1	0	1	0	4 Fair
Chang et al. (2019); Taiwan (23)	1	1	0	0	1	0	1	1	5 Fair
Hong et al. (2018); South Korea (24)	1	1	0	0	1	0	1	1	5 Fair
Chu et al. (2019); China (25)	1	1	0	0	1	0	1	0	4 Fair
Chang et al. (2017); Taiwan (26)	1	1	0	1	1	0	1	1	6 Fair
Chen & Chen (2020); China (27)	1	1	0	0	1	0	1	1	5 Fair
He et al. (2022); China (28)	1	1	1	1	2	1	1	1	9 Good
Yang et al. (2022); China (29)	0	1	1	1	2	0	1	1	7 Good
Xiong et al. (2023); China (30)	1	1	1	1	1	0	1	1	7 Good
Li et al. (2023); China (31)	1	1	1	0	2	0	1	1	7 Good
Yuan et al. (2021); China (32)	1	1	1	0	1	1	1	1	7 Good
Ren et al. (2023); China (33)	1	1	1	1	0	1	1	1	7 Good
Yan et al. (2023); China (34)	0	0	1	0	0	0	1	1	3 Poor
Gao et al. (2021); China (35)	0	1	1	1	2	0	1	1	7 Good
Perret et al. (2021); South Korea (36)	1	1	1	0	2	0	1	1	7 Good
Yang et al. (2023); China (37)	1	1	1	0	2	1	1	1	8 Good
Liang et al. (2023); China (38)	1	1	1	0	2	0	1	1	7 Good
Shen et al. (2023); China (39)	1	1	1	0	2	0	1	1	7 Good
Long, Zhou & Li (2020); China (40)	1	1	0	1	2	0	1	1	7 Good
Yu et al. (2023); China (41)	1	1	0	1	1	0	1	1	6 Fair
Zhao & Li (2022); China (42)	1	1	0	1	2	0	1	1	7 Good

### 3.1 Prevalence rates and patterns of bullying

**Table 4** summarizes prevalence of bullying amongst adolescents in East Asian countries. The most commonly used bullying questionnaire was the adapted, revised, or translated version of Olweus Bully Victim Questionnaire (OBVQ), used in 25 studies.

**Table 4:** Prevalence of Bullying and Victimization.

Author (Year); Country [Ref]	Traditional bullying victimization	Cyberbullying victimization	Traditional bullying perpetration	Cyberbullying perpetration	Bully-victims
Kawabata et al. (2014); Taiwan (22)	Mean=0.03, SD 0.90 (relational); Mean=0.04, SD 0.94 (physical) at baseline	NR	NR	NR	NR
Chang et al. (2019); Taiwan (23)	NR	NR	NR	NR	NR
Hong et al. (2018); South Korea (24)	6.10%	NR	NR	NR	NR
Chu et al. (2019); China (25)	NR	NR	NR	NR	NR
Chang et al. (2017); Taiwan (26)	Mean=1.22; SD 0.43 (male); Mean =1.27; SD 0.46 (female)	NR	NR	NR	NR
Chen & Chen (2020); China (27)	NR	NR	NR	NR	NR
He et al. (2022); China (28)	NR	NR	NR	NR	NR
Yang et al. (2022); China (29)	NR	NR	NR	NR	NR
Xiong et al. (2023); China (30)	NR	NR	NR	NR	NR
Li et al. (2023); China (31)	Baseline” 61.3%	Baseline: 15.3%	NR	NR	NR
Yuan et al. (2021); China (32)	NR	NR	NR	NR	NR
Ren et al. (2023); China (33)	NR	NR	NR	NR	NR
Yan et al. (2023); China (34)	NR	NR	NR	NR	NR
Gao et al. (2021); China (35)	NR	NR	NR	NR	NR
Perret et al. (2021); South Korea (36)	Peer victimization: 8.2% -Boys (11.3%); girls (5.1%) Girls twice as likely to report depressive symptoms when victimized	NR	NR	NR	NR
Yang et al. (2023); China (37)	NR	NR	NR	NR	NR
Liang et al. (2023); China (38)	NR	NR	NR	NR	NR
Shen et al. (2023); China (39)	Baseline: 33.6%	NR	NR	NR	NR
Long, Zhou & Li (2020); China (40)	NR	NR	NR	NR	NR
Yu et al. (2023); China (41)	Victims group T1 = 12.93% T2 = 15.55%	NR	NR	NR	Bully Victims group (both perpetrated and were victims as well) T1 = 18.34% T2 = 14.01%
Zhao & Li (2022); China (42)	NR	NR	NR	NR	NR
Chan (2013); China (43)	14% (poly-victimization = exposure to high levels and multiple types of victimization i.e. physical, sexual, peer, neighborhood violence) 4 or more types of victimization Females=11.7% Males=16.2%	NR	NR	NR	NR
Chang et al. (2015); Taiwan (44)	NR	30% (Internet addiction group); 12.9% (non-Internet addiction group)	NR	24.2% (Internet addiction group); 7.8% (non-Internet addiction group)	NR
Chang et al. (2013); Taiwan (45)	8.2% Females=7.4% Males=8.9%	18.4% F=17.2% M=19.6%	10.6% F= 6.1% M=14.7%	5.8% F=4.5% M=7.0%	5.1% F=2.2% M=7.7% (traditional); 11.2% (cyber) F=5.8% M=16.3%
Chen et al. (2020); Taiwan (46)	NR	NR	NR	NR	NR
Chen et al. (2018); China (47)	28.9% Females=13.0% Males=15.9%	3.7% F=1.1% M=2.6%	NR	NR	NR
Chen et al. (2018); Hong Kong (48)	NR	15-31% for doxing	NR	NR	NR
Chu et al. (2018); China (49)	NR	74.6%	NR	NR	NR
Guo et al. (2020); China (50)	42.41% (51.5% of boys and 34.3% of girls)	NR	NR	NR	NR
He et al. (2019); China (51)	NR Peers use of emotional bullying Females=37.9% Males=50.8%	NR	NR	NR	NR
Hong et al. (2018); South Korea (52)	NR	NR	NR	NR	NR
Hong et al. (2016); China (53)	4.5%	NR	1.5%	NR	3.0%
Hu et al. (2016); Taiwan (54)	20.2%	NR	NR	NR	13.9%
Jung et al. (2014); South Korea (55)	3.4%	3.3%	NR	NR	3.0%
Kozasa et al. (2017); Japan (56)	19.2%	NR	6.6%	NR	22.8%
Lee & Kim (2017); Korea (57)	NR	NR	NR	NR	NR
Li et al. (2018); China (58)	28.2%	NR	NR	NR	NR
Ma et al. (2018); Taiwan (59)	Mean=2.06 (SD 0.55)	NR	NR	NR	NR
Min et al. (2015); South Korea (60)	NR	NR	NR	NR	NR
Seo et al. (2017); South Korea (61)	8.2% (10.6% of females and 6.4% of males)	NR	NR	NR	NR
Shao et al. (2014); China (62)	27.4%	NR	NR	NR	NR
Tang et al. (2018); China (63)	18.5% (Left Behind Children (LBC)); 11.3% (control) Include the occasionally bullied 48.3% LBC, 44% control	NR	NR	NR	NR
Wang et al. (2019); China (64)	NR	NR	NR	NR	NR
Wang et al. (2020); China (65)	NR	NR	NR	NR	NR
Xiong et al. (2019); China (66)	Mean=0.68, SD 0.48 (males); Mean=0.62, SD 0.46 (females)	NR	NR	NR	NR
Yeh et al. (2019); Taiwan (67)	34.8% (verbal and relational bullying); 15.0% (physical bullying)	NR	NR	NR	NR
Yen et al. (2014a); Taiwan (68)	25.0% (21.4% passive bullying and 8.4% active bullying)	19.6% (17.7% passive bullying and 5.5% active bullying)	NR	NR	NR
Yen et al. (2014b); Taiwan (69)	Mean=2.1; SD 2.0 (passive bullying); Mean=0.6; SD 1.2 (active bullying)	NR	NR	NR	NR
Yen et al. (2014c); Taiwan (70)	NR	19.1%	NR	14.3%	NR
Yin et al. (2017); China (71)	Mean=1.54; SD 0.70	NR	NR	NR	NR
Yun & Kim (2016); South Korea (72)	23.5% for all types of bullying including cyberbullying (31.8% of girls and 14.7% of boys) 18.1% (relational); 12.8% (verbal); 3.5% (physical); 2.8% (property-related)	5.6%	22.5% for all types of bullying including cyberbullying (27.2% of girls and 17.5% of boys) 10.6% (relational); 13.8% (verbal); 4.6% (physical), 1.5% (property-related)	NR	NR
Zhou et al. (2021); China (73)	NR	NR	NR	NR	NR
Pan & Spittal (2013); China (74)	25.4% Total (0.92% for racial or religious bullying)	NR	NR	NR	NR
Zhao et al. (2021); China (75)	SSA (same sex attraction) Males 23%, Females 24% BSA (both sex attraction) Males 26.5%, Females15.5% OR 1.47 (Males), 1.38 (Females)	SSA M 18% F9.6% BSA M 10.9% F11.6% OR 1.77 (M) 1.6 (F)	NR	NR	NR
Mei et al. (2021); China (76)	NR	NR	NR	NR	NR
Lai et al. (2023); China (77)	Verbal 11.2% Relational 5.5% Physical 3.2%	1.7%	NR	NR	NR
Liu et al. (2020); China (78)	Sibling Bullying Verbal 13.8% Relational 4.0% Physical 7.6%	NR	Sibling Verbal 14.5% Relational 2.8% Physical 6.5%	NR	NR
Guo et al. (2022); China (79)	NR	NR	NR	NR	NR
Peng et al. (2022); China (80)	12.5% sibling bullying 10.1% peer bullying 4.7% both sibling and peer bullying Lesbian, gay, bisexual, transgender, queer (LBGTQ) have higher OR of sibling (OR 1.41, 95%CI 1.08-1.85), peer (OR 1.54, 95%CI 1.13-2.08), both sibling & peer (OR 2.23 95% CI 1.48-3.34)	NR	NR	NR	NR
Zhu et al. (2020); China (81)	- Peer or sibling assault 11.51% one time; 5.93% two or more - Bullying 6.51% one time; 4.28% two or more time of any type of bullying Emotional bullying 10.09% one time; 7.62% 2 times or more	Sexual – passing on nude pictures 1.08% 2 or more times	NR	NR	NR
Wen et al; China (2022) (82)	NR	NR	NR	NR	NR
Zhu et al. (2021); China (83)	NR	NR	NR	NR	NR
Cao et al. (2021); China (84)	NR	NR	NR	NR	NR
Liu et al. (2023); China (85)	12.5% (8.9% traditional; 3.6% both traditional and cyber)	6.9% (3.3% cyber + 3.6% both traditional and cyber)	NR	NR	NR
Fan et al. (2021); China (86)	NR	NR	NR	NR	NR

NR, Not reported.

For the 65 studies that were included, the sample size ranged from 194 to 18,341 participants. Of these, 3 studies were based on a large population study in children from 2009 – 2010 (n = 18,34) (43, 47, 81); and 2 studies were based on the Korean Child Youth Panel Survey (KCYPS) (n = 2,283) (36, 57).

Prevalence of bullying was reported as either in the preceding 6- or 12-month period, lifetime, or not reported. Twenty-three studies reported on the preceding 12-month prevalence which ranged from 6.1% to 61.3% in traditional bullying victimization and 3.3% to 74.6% in cyberbullying victimization. Lifetime prevalence of bullying victimization ranged from to 8.2% to 71.4%.

Rates of bullying victimization trended highest in the rural areas (range: 18.5 – 49.8%; average: 46%), intermediate in studies with urban and rural participants (range: 8.2 – 61.3%; average: 28%) and lowest in urban areas (range: 4.5 – 74.6%; average: 21.9%).

Children and adolescents who were perceived as ‘different’ to peers experienced higher rates of bullying, including children with Attention Deficit Hyperactivity Disorder (ADHD) (up to 49.8%) (45), Left Behind Children (LBC- children who are left behind when parents go to work) (up to 48.3%) (63) in rural studies, and those in the sexual minority (up to 26.5%) (75, 81). Males experienced more traditional bullying victimization than females (36, 43, 45, 47, 50, 51, 63, 75) except in one study (61) with higher rates in females (10.6% in females vs. 6.4% in males) (**Table 4**). Females also tended to experience more relational bullying while males experienced physical bullying more (72, 74) (**Table 4**).

Twelve papers addressed cyberbullying victimization in the preceding 12 months with prevalence rates (median: 18.6%) ranging from 3.3% (55) in South Korea to 74.6% (49) in Wuhan, China. Rates of cyber-victimization and cyberbullying more than doubled to 30% if the victim or bully had an internet addiction (44). One-fifth (22.2%) of Japanese cyberbullies were victims themselves i.e., cyberbully-victims (56). Cyberbullying rates were either higher in females (48, 56), or approaching those of male rates (17.2% in females vs. 19.6% in males) (45). In 4 studies, bullying victimization occurred more in older children (23, 31, 49, 71), while it was higher in younger children in only one study (43).

### 3.2 Depression assessment

The most commonly used depression scales were adaptions or translations of the Center for Epidemiological Studies Depression (CES-D) and Center for Epidemiological Studies Depression Scale for Children (CES-DC) (n = 28 studies).

### 3.3 Risk and protective factors for bullying and depression in East Asian adolescents

Many studies showed a correlation between peer victimization and the presence of depression or depressive symptoms in adolescents in East Asian cultures (24, 26, 45, 49, 51, 58, 59, 64, 65). In Taiwan, bully-victims reported the highest odds of depression (β = 8.46***) and lowest levels of self-esteem compared to the other two groups of victims only (β = 6.51***) or bully only (β = 1.23*) (68). Factors to be considered in bullying and depression are described below; whether they increased risk of bullying or depression or whether they were protective.

Results are presented as individual, family and community factors as well as vulnerable groups for comparison and description of the themes across the various studies (**Table 5**). Details of statistics, sample size, correlations are included in **Table 5**. Studies of “good” quality and low risk of bias (described as NOS score ≥ 7) with larger sample sizes (n > 1,000) are emphasized.

**Table 5:** Factors associated with bullying and/or depression.

Author (Year); Country [Ref]	Individual factors (i.e., age, gender, traits)	Family factors (family functioning, siblings)	Community factors (peers, friendships, teachers, schools)	(V) Variables CoV (CoVariates) included in analysis # Bullying and depression association Main findings	Statistical Analysis	NOS score
Kawabata et al. (2014); Taiwan (22) (n = 389)	Depressive symptoms predicted traditional relational bullying victimization in highly interdependent children	NR	NR	(V) – peer victimization, Relational-interdependent construals, depression At T1, victimization was (+) associated with depression (r=0.16-0.38*). At T1 relational (but not physical) victimization (+) predicted depressive symptoms at T22 (6 months later) for highly interdependent children (r=0.72, p<0.001).	CFA (confirmatory factor analysis with good model fits	4
Chang et al. (2019); Taiwan (23) (n = 4,072)	NR	NR	NR	V = Sleep problems, peer victimization, depressive symptoms, age, sex CoV – parent education Sleep problems were (+) and significantly*** associated with depressive symptoms in females but not in males after controlling for covariates) (+)	Mediation analysis with PROCESS	5
Hong et al. (2018); South Korea (24) (n = 1,750)	NR	NR	NR	V = Depression, Peer Victimization, Supporting parenting CoV= demographics Adol. With Lower family income, past experience of peer victimization (b=0.137***), less supporting parenting were more likely to report higher levels of depression compared to peers.	GEE – generalised estimating equation	5
Chu et al. (2019); China (25) (n = 661)	Boys (n=401@T_1_) were more likely than girls (n=260) to experience TBV (F_(1,594)_ =8.47**) and cyBV (F_(1,594)_ = 4.24*) Older adolescents had greater symptoms of depression (F_(1,594)_=4.13*) and general anxiety (F_(1,594)_=3.87,p=0.05),stress(F_(1,594)_=4.43*),when comparing Grade 7 to Grade 8	NR	NR	V – TBV, CyBV, Depression, anxiety, stress, Self-esteem, social anxiety, Loneliness Adol. with psychosocial problems were more likely to experience BV. Depression and general anxiety was a predictor of TBV and CyBV. Stress was a predictor of TBV. Self-esteem, social anxiety, and loneliness was a predictor of CyBV.	Cross-lagged analysis With good model fits	5
Chang et al. (2017); Taiwan (26) (n = 1,893)		NR	NR	V – BMI, peer victimization, sleep problems, depressive symptoms CoV – demographics Higher BMI predicted more peer victimization, leading to more sleep problems, and to higher levels of depressive symptoms. Total effect on N=1893 b -0.039, SE 0.02, (95%CI - 0.079 to 0.001) b – standardized regression co-efficient	Multiregression models with bootstrap approach	6
Chen & Chen (2020); China (27) (n = 1,507)		NR	NR	#Group level Depression (+) predicted individual level Depression, (+) predicted later peer victimization. N=1430 Effect of T_1_ group level depression on T_2_ adjustment variable of Peer victimization Effect 0.41, SE0.17, t value 2.47* 95%CI (0.8,0.74)	Multilevel analysis	6
He et al. (2022); China (28) (n = 1,687)	Boys (n = 1,019) scored higher at T_1_(M= 10.12 SD 2.91) and T_2_ (M= 9.18 SD 2.27) than girls (n = 668) at T_1_(M=9.44 SD 2.23) and T_2_(M=8.84 SD 1.85) on bully victimization where the mean difference was 0.68*** at T_1_ and 0.34*** at T_2_	NR	Among girls bullying victimization significantly predicted poor quality of sleep** and depressive symptoms*** at T2. #In both boys and girls, severe depressive symptoms significantly predicted more victimization from T_1_ to T_2_ *and sleep problems and were (+) associated with depressive symptoms.	V -Depressive symptoms, BV,BP, Sleep quality CoV – demographics -gender, family structure, academic record etc Bidirectional association b/w school bullying, depressive symptoms and sleep problems.	Cross lagged autoregressive models in SEM with good model fits	9
Yang et al. (2022); China (29) (n = 450)	T_1_ bullying→T_2_ self esteem→T_3_ Depressive symptoms The effect of T_2_ self-esteem on T_3_ depressive symptoms was significant (β= -0.369, SE 0.056 t=-6.588, p<0.001 LLCI -0.480 to -0.259)	Higher parent educational levels→Lower level of depressive symptoms Higher parent educational levels→ higher self-esteem@T_2_ (β=0.381 SE 0.173 t=2.203 p=0.028 LLCI 0.041 to ULCI 0.721)	Girls had higher levels of friendship intimacy and depressive symptoms T_1_ BV and T_1_ Friendship intimacy is a moderator with a (+) correlation with T_2_ self-esteem (β=0.090, t=2.269, p=0.024 LLCI 0.012 to 0.168 ULCI) but not the relation between T1 BV and T3 depressive symptoms with symptoms (B=-0.028 SE 0.052 p=0.586)	V – depression, BV, self-esteem, friendship intimacy #Peer victimization was assoc with depression (r=0.58*) T_1_ BV was (+) predicted T_3_ depressive symptoms (β=0.156 SE0.056 t=2.789 P=0.006 LLCI 0.046 to ULCI 0.267) when T1 friendship intimacy was low The association was weakened by solution-oriented conflict resolution strategies (B=-0.35, p<0.01). This serves as an adaptive strategy in adolescents. The association was strengthened by non-confrontational strategies in girls (B=0.18, p<0.01) despite other research suggesting that this was an adaptive strategy.	OLS (ordinary least squares) regression based path analysis similar to SEM (structural equation modelling)	7
Xiong et al. (2023); China (30) (n = 2,551)	Where n=2613@T_1_ T_1_ Personal BJW mediates the association between T_1_bullying victimization and T_2_ depressive symptoms. T_1_ BV was negatively related to T_1_ personal BJW (γ = -0.27, SE = 0.03, *p <*0.001) T_1_ Personal BJW was negatively related to T_2_ depressive symptoms (γ = -0.03, SE = 0.01, *p* < 0.05) Decreased personal Belief in a just world led to more depressive symptoms	NR	T_1_ personal BJW was moderated by T_1_classroom-level victimization; with stronger effect for adolescents in classrooms with low levels of victimization.	Variable – General and Personal belief in a just world (BJW), BV, Depressive symptoms	Multilevel moderated mediation model (MSEM)	7
Li et al. (2023); China (31) (n = 10,279)	TBV Bullying victimization scores higher for: - higher (slightly) for Males (n=5089) M=11.7(5.0) Vs 10.0 (3.2)*** -Older adolescents (N=2498) >=16 yrs (M=11.3(4.4) Vs 15 yrs M10.8 (4.3), <= 14 yrs M=10.4 (3.8),*** -those with low cognitive ability scores (n=2631) M=11.6 (5.0) vs 10.4(3.9) ***with highest ability scores (n=2210)	Traditional BV scores higher for those who: -have siblings (n=6774) M=11.0 (SD 4.2) Vs M=10.5 (SD4.4) *** with no siblings -not living with parents (n=3084) M=11.3 (4.4) vs 10.6 (4.2)*** -Not close to parents (n=5335) M=11.3 (4.4) vs 10.6 (4.2)*** -	BV scores higher for Rural status (n=7196) M=11.0(4.3) vs Urban M=10.4(4.1)*** Traditional BV scores higher for those who: -Relatively low parental education levels n=3249 M=11.0 (4.2) vs M=10.6 (4.3)*** -And Low parental education expectations (N=3249 M=11.3(4.8) vs 10.6 (4.0)*** -Worse family financial conditions (N=2385 11.9(5.2) vs 10.9 (3.9)***	V – TBV, CyBV, Depressive Symptoms CoV – Demographics Mediators – physical health, healthy lifestyles, sleep quality, academic achievement TBV and CyBV predicted depressive symptoms at 2*** and 5 year*** follow up Being bullied predicted worse healthy lifestyles, poorer sleep quality and lower academic achievements*, all correlated with development of depressive symptoms***	SEM, multigroup analysis	7
Yuan et al. (2021); China (32) (n = 1,390)	Higher levels of cyBP (r=0.288***) predicted lower levels of mindfulness(r=-0.495***) and severe depressive symptoms(r=0.623***)	NR	NR	V – CyBV, Mindfulness, Depression Cyberbullying, mindfulness and depression would predict each other over time	Exploratory Factor Analysis; Pearsons correlation;Cross lagged model; SEM	7
Ren et al. (2023); China (33) (n = 1,911)	NR	NR	NR	Being ‘threatened or intimidated’ was the most influential bullying behavior within victimization items. Being robbed or blackmailed was the most influential item on adol depressive symptoms.	Graphical Gaussian Model	7
Yan et al. (2023); China (34) (n = 592)	NR	NR	NR	V – Child Maltreatment, BV, Negative thoughts, Self-compassion, Depression, NNSI (non suicidal self injury) Child Maltreatment (+) affects depression and (NSSI) directly and through negative thoughts. BV affects depression and NSSI	SEM	3
Gao et al. (2021); China (35) (n = 2,407)	When compared to males (n=1191), females had significantly less cyBV at T1, t(2128.56)=5.54***; cyBV at T2, t(1919.23)=4.93***	NR	Peer pressure significantly mediated the relationship between cyBV at T1 and depressive symptoms at T2.	V - CyBV, Depressive symptoms, Peer pressure	SEM	7
Perret et al. (2021); South Korea (36) (n = 2,258)	Peer victimization: Higher prevalence rate in boys n=1139 (11.3%) than in girls n=1118 (5.1%)*** Boys reported lower levels of friend support (M16.08+-3.04 Vs M15.74(+/-3.79) p=0.017), fewer depressive symptoms (10.76+/-6.29 Vs 8.89+/-6.06*** than girls	NR	Higher levels of friend support attenuated the association between peer victimization*and depression symptoms.	V – Peer victimization, depressive symptoms, perceived friend support, Prior mental health symptoms, sociodemographic, family factors Significant difference in depressive symptoms between victimized and non-victimized in low***and moderate friend support***. No difference in depressive symptoms between victimized and non-victimized in high friend support (p=0.73) Peer victimization was associated with MORE depressive symptoms for adols with low and moderate friend support, but not those with high friend support.	Linear regression model	7
Yang et al. (2023); China (37) (n = 2,339)	4 ‘profiles’ 1.Normative n=1384 – lowest levels of BV, lowest level of depression, highest subjective well being 2.’Vulnerable’ n=678 29%) - low levels BV, higher level depression, lower subjective well-being 3. ‘Resilient’ n=188(8%) – higher levels BV, low level of depression, high levels of subjective well being 4. ‘Adverse’ n=89 (3.8%) = highest level of BV, highest level depression, lowest level subjective well- being	NR	Adolescents in the ‘Resilient’ profile benefitted from more teacher and peers support. -Support from teachers and peers (+) correlated with subjective well-being r=0.28***; r= 0.31***) -Correlated (–) with bullying victimization (r= -0.20***; r=-.0.51***) -Correlated (–) with depressive symptoms (r= -0.23***; r=-0.30***)	V -BV, Depressive, subjective well being, support of teachers and peers, demographics The presence of a ‘resilient’ profile was identified with 3 other distinct profiles. Adolescents with lower levels of both teacher support (b=-0.87**, OR=0.42) and peer support (b=-1.05**, OR=0.35 were more likely to be classified as ‘Adverse’ profiles, rather than the ‘Resilient’ profile	Latent profile analysis (LPA), LR	7
Liang et al. (2023); China (38) (n = 3,510)	NR	NR	NR	Study examined profiles and transitions of NSSI and depressive symptoms with predictive role of BV in subgroup memberships and transitions. Adol in at-risk profiles showed varying degrees of transition	LPA	7
Shen et al. (2023); China (39) (n = 1,205)	NR	NR	NR	Peer victimization was (+) associated with depressive symptoms.	Cross lagged panel model	7
Long, Zhou & Li (2020); China (40) (n = 447)	Adolescents’ relational victimization positively correlated with popularity status insecurity (r=0.22 for the low popularity group and r=0.17 for the high popularity group, ps<0.01). Adol with lower popularity had significantly higher scores in -relational victimization t(205.83)=5.14*** -anxiety t(324.08) = 2.33* Depressive symptoms t(331.51)=3.21** at T_1_	NR	NR	V – relational victimization, Popularity status insecurity, Popularity status, Depressive symptoms, anxiety Popularity status insecurity mediated the longitudinal associations between relational victimization and depressive symptoms and anxiety for adolescents with relatively low popularity.	SEM	7
Yu et al. (2023); China (41) (n = 1,711)		Adolescents with higher levels of parent psychological control were more likely to be BV or bully-victims (B-V) (OR=1.35, p=0.1 95%CI (1,07,1.70); (OR 1.36** 95%CI (1.11,1.65) compared to those with lower levels of parents control	Higher friendships quality decreased risk of being a victim or bully victim (OR=0.54*** 95%CI (0.39,0.76); (OR=0.52*** 95%CI (0.38,0.70) Higher quality friendships increased odds of moving from a victim or bully-victim to non-involved adolescent [Victim to Uninvolved (OR 7.31***(3.59,14,88)]; [Bully-victim to Uninvolved (OR17.07***(8.05, 36.20)	V - School BP & BV, Depression, Friendship quality, Parental psychological control Adolescents with higher levels of depressive symptoms were more likely to be victims or bully-victims	LPA	6
Zhao & Li (2022): China (42) (n = 691)	NR	NR	Physically and relationally (but not verbally) victimized adolescents in healthy cliques with lower victimization norms reported committing more reactive (not proactive) forms of aggression (externalization)(B_physical victimization_ =-0.10, SE 0.04*; B _relational victimization_ =0.10, SE=0.04* and having more depressive symptoms 2 years later (internalization) B_physical victimization_ =0.20, SE 0.07**. This is consistent with the “healthy context paradox”	V - Peer cliques(T1), Peer victimization (T1), Clique victimization norms (T1), Reactive and proactive aggression (T1 and T2), Depressive symptoms (T1 and T2)	Multilevel model analysis	7
Chan (2013); China (43) (n = 18,341)	Age_M_ negatively assoc with BV (AOR 95%CI 0.91***(0.88,0.95) Male gender (n=9776) assoc with BV (AOR 95%CI 1.19***(1.11,1.28)	Having siblings was associated with bullying victimization (AOR 95%CI 2.00***(1.85, 2.16)	Living in cities in Mainland China associated with BV. (AOR 95%CI 1.81***(1.66, 1.98)	V - Child victimization, PTSD, Depression, Health related QOL, deliberate self harm and suicide ideation -PTSD (+) assoc with BV (AOR 95%CI 1.09***(1.04,1.15) -Deliberate self harm and suicide ideation (+) assoc with BV (AOR 95%CI 1.38***(1.26,1.52) -Higher Physical health (i.e better well being) and higher Mental health scores (-) assoc with BV (Physical Health (AOR 95%CI 0.91***(0.88,0.95), Mental Health (AOR 95%CI 0.99***(0.99,1.00)	2 phase LR	8
Chang et al. (2015); Taiwan (44) (n = 1,867)	Male gender (n=901) assoc with cyBV (OR 1.62 95% CI 1.23,2.13) and cyBP (OR 1.59 95% CI 1.14,2.22) Poor academic performance assoc with cyBV (OR 1.05 95% CI 0.77,1.44) and cyBP (OR 1.09 95% CI 0.75,1.58)	Having lower parental attachment(OR 0.79 95% CI 0.68,0.93), fewer parental restrictions(OR 0.89 95% CI 0.82,0.95), was associated with assoc with cyBV Adol with lower parental attachment more likely to experience internet addiction, CyBV, smoking, depression Adols with higher parental restrictions had less Internet addiction and CyBP		V – Online activities, internet addiction, Parental mediation, Parental attachment, CyBV/CyBP, Tobacco & alcohol use, self-esteem Poor academic performance assoc with cyBV (OR 1.05 95% CI 0.77,1.44) and cyBP (OR 1.09 95% CI 0.75,1.58) Assoc with cyBV (n=1787): -lower internet literacy OR 0.53 95% CI 0.41,0.68) -Internet addiction assoc with cyBV(OR 1.82 95% CI 132,2.52) and cyBP(OR 2.13 95% CI 1.47,3.07).	Multiple LR	7
Chang et al. (2013); Taiwan (45) (n = 2,992)	Male gender more likely to be cyBP (OR1.57 95%CI=1.15,2.16) and cyB-V (OR 3.17, 95%CI 2.44-4.10) than females. cyBV (β=-0.62 SD0.24 p=0.009) and cyB-V(β=-0.67 SD0.31 p=0.029) more likely to have lower self-esteem than non involved.	NR	School BV(β=-1.25 SD0.33 p=<0.001) and School B-V (β=-1.53 SD0.44 p=<0.001) were more likely to have lower self-esteem and higher depression than other groups	V – School BV/BP, self-esteem, depression, demographics Students with lower academic performance were more likely to be school B-V(n=151) (OR= 2.05, 95%CI 1.47-2.86) and cyB-V(OR 1.69, 95%CI 1.34-2.15) Students from Lower social-economic status more likely to be victims only (n=244) (OR 1.64, 95%CI 1.23-2.09) Students with Internet risk behaviours more likely to be school bully-victims (n=151) (OR 1.98, 95%CI 1.31-2.99) or cyberbully-victims (n=336) (OR 4.28, 95%CI 3.00-6.10)	Univariate LR	7
Chen et al. (2020); Taiwan (46) (n = 2,419)	NR	NR	School victimization by students (r=0.26**) and by teachers(r=0.16** BOTH correlated positively with adolescent depression and marginally with parental depression (for victimization by students r=0.07**; for victimization by teachers r=0.04*. Parental depression and adolescent depression were positively correlated (r=0.16**).	V= gender, age, BV by students, BV by teachers, Adolescent and parental depression Overall, adolescent depression was a significant predictor of victimization by students and victimization by teachers The model explained school victimization better for males than for females for both victimization by students and victimization by teachers.	CFA with good model fits; SEM	7
Chen et al. (2018); China (47) (n = 18,341)	Boys (n=9678) were more likely to be cyBV (AOR=2.73*** (2.179,3.411) and TBV(AOR=1.23***(1.129,1.336) than girls	Parental divorce/separation/parent widow status(AOR =1.37-1.68*), having siblings (AOR =1.36*-1.41***) were associated with BP All types of Family victimization (conflicts within family) were associated with greater risk of BV (AOR =1.99-5.36***). All types of family victimization except neglect were associated with cyBV (AOR =2.24-5.36***)		V – demographic, CyBV and TBV, Family violence, PTSD, Depression, Health related QOL, Deliberate self harm and Suicide ideation Below-median family income (AOR =1.11-1.35*), low maternal education level (AOR =1.37-2*), paternal unemployment (AOR =1.43-2.13**) associated with BP. OR’s are higher for internet victimization than on-internet except for children with siblings Significant assoc between BV and all health correlates, PTSD, depression, physical and mental health and suicide ideation (AOR=0.95-1.54*)	2 phase regression analysis Multinomial LR and LR	7
Chen et al. (2018); Hong Kong (48) (n = 2,120)	Girls were more likely than boys to be victims of doxing.	NR	NR	V – demographic, Depression,Anxiety, Stress, experience of doxing 1) Almost all types of disclosed personal information results in negative feelings, including depression, anxiety, and stress. 2) Significant associations were found between i) emotional problems and the disclosure of mobile phone numbers, personal photos and videos. ii) doxing conducted by schoolmates and anxiety and depression.iii) doxing through Instant Messenger and anxiety.	Spearman’s coefficient values	5
Chu et al. (2018); China (49) (n = 489)	Older students reported higher levels of cyberbullying victimization. Self-compassion moderated the association between cyBV and hopelessness and depression. For low self compassion individuals (one SD below the mean) CyBV was associated with hopelessness (β_simple_=0.70,***) and depression (β_simple_ =0.36*). For high self-compassionate individuals (one SD above the mean), the effects of CyBV on hopelessness and depression were weak (β_simple_ =0.31**) and non significant (β_simple_ =-0.10p=0.4)	NR	NR	V – CyBV, hopelessness, self-compassion,depression, anxiety CyBV (+) predicted hopelessness. Hopelessness (+) predicted depression and anxiety Hopelessness partially mediated the relation between cyberbullying victimization and depression & anxiety (β = 0.18, p < 0.001) High self-compassion was protective (Low self compassionate individuals associated with hopelessness and anxiety from cyberbullying victimization) #Older students tended to report higher levels of cyberbullying victimization and depression, as well as lower levels of self-compassion. Cyberbullying victimization was positively associated with hopelessness, depression, and anxiety The direct relationship between cyberbullying victimization and depression was significant (β = 0.20***)	Pearson Correlation, Linear LR	6
Guo et al. (2020); China (50) (n = 1,252)	NR	NR	(1) High school students with more friend support and school support had lower levels of depressive symptoms. (2) School support reduces depressive symptoms in girls who have been victimised (ß −0.20, 95% CI −0.34–0.06)	V- Depression, BV, social support, Variables controlled including demographic High school students who had been bullied had much higher depressive symptom scores than those where had not (beta [ß] = 1.43, 95% confidence interval [CI] 0.95–1.91)	Multivariable Linear LR	8
He et al. (2019); China (51) (n = 6,576)	NR	NR	Compared to adol with no experience of punishment, Depressed Males had: - more teachers’ use of emotional punishment (β=1.40 95% CI (1.063,1.746)*** -more teachers’ use of physical punishment (β=1.862 95%CI(1.442,2.283)*** -less school connectedness (β=-0.34 95%CI(-0.38,-0.308)***	V – demographic, depressive symptoms, Conflicts with father and mother, conflicts with teachers, conflicts with peers, school connectedness Depressive symptoms were (+) associated with -Quarrel with peers (β=1.738 95%CI (0.88, 2.60***).-Fight with peers (β=1.738 95%CI (0.88, 2.60***). -peers use of emotional bullying (β=1.46 95%CI (0.62, 2.29***). And Negatively associated with School connectedness (β=-0.448 95%CI (-0.58, -0.35***)	Multiple LR	7
Hong et al. (2018); South Korea (52) (n = 10,453)	Male gender (n=5831) associated with direct cyBV (β=0.287***, 95%CI 0.239,0.334)	high levels of parental abuse (β=0.05***, 95%CI 0.033,0.069); high levels of parental neglect (β=0.025*, 95%CI 0.003,0.047); and high levels of family dysfunction (β=0.053**, 95%CI 0.02,0.086) were associated with direct cyBV	Poor peer relationships (β=0.037*, 95%CI 0.006,0.067); Higher teacher abuse (β=0.085***, 95%CI 0.069,0.102); and high school victimization levels (β=0.197***, 95% CI 0.184,0.209) were associated with direct cyBV. Higher Neighbourhood safety the less likely involvedment with direct cyBV (β= -0.069***, 95%CI -0.098,-0.040)	V- Direct and indirect CyBV, family variables i.e parent abuse, parent neglect, family dysfunction, respectful peer, poor peer relationship, school variable i.e teacher abuse,school victimization, school connectedness, neighbourhood safety, economic hardship Parent neglect was related to indirect CyBV, while parent abuse, parent neglect and family dysfunction were assoc with direct CyBV.	Linear LR	7
Hong et al. (2016); China (53) (n = 20,511)	NR	NR	NR	V – Suicidal ideation, BP, Depression, demographic A significant interaction was identified between bullying and depression in both unadjusted and adjusted models (P-interaction = 0.001 in both models) suggesting that depression may modify the influence of bullying on SI (suicidal ideation)	Univariate and multivariate LR	6
Hu et al. (2016); Taiwan (54) (n = 287)	NR	NR	NR	V – Anxiety symptoms, Depression symptoms, ADHD features, BP, Behavioural termperament traits, ASD ADHD: severity of inattention symptoms was (+)associated with the severity of depression after controlling for the effects of sex and age. ADHD adolescents with higher behavioral inhibition system (BIS) score (but not BAS), comorbid ASD, or were bullying victims had more severe anxiety and depressive symptoms # Being bullying victims (β = 0.190, t = 3.454, p = 0.001) or perpetrators (β = 0.228, t = 4.308, p <0.001) were positively associated with severity of depression; ADHD adolescents who were bullying perpetrators reported more severe depressive symptoms than those who did not bully others	Multiple Regression analysis	7
Jung et al. (2014); South Korea (55) (n = 4,531)	Boys had a higher prevalence of PIU compared to girls (16.1% vs 8.1%) All groups including: -victim only (OR (95% CI): 2.36 (1.58-3.54)***.-bully only (OR (95% CI): 1.66 (1.09-2.53), p=0.018)-bully-victim group (OR (95% CI) 2.38(1.58-3.60)*** had a higher likelihood of PIU than the neither victimised nor bully group	NR	NR	V – CyBV, Problematic internet use, depression and other psychopathology, age,gender, grade Cyberbullying victims were also more likely to be depressed (OR (95% CI): 4.2 (2.11-8.35***)	LR	7
Kozasa et al. (2017); Japan (56) (n = 827)	Boys who are B-V (n=35) have higher mean scores compared with the neither group (n=146) in -social problems (M_B-V_=5.06 (SD2.81) Vs M_Neither_=2.75(SD2.25)*** -attention problems (M_B-V_=7.29 (SD2.44) Vs M_Neither_=4.8(SD3.11)*** -aggressive behaviour, (M_B-V_=9.80 (SD5.39) Vs M_Neither_=5.68(SD4.30)*** -externalizing scale(M_B-V_=12.23 (SD6.20) Vs M_Neither_=7.40(SD5.81)***. For adolescent girls, the mean scores for the B-V group were significantly higher than those for the Neither group for all the dimensions and subscales measured above.	NR	NR	V – questions on BV and BP, Youth Self Report items For preadolescent girls, the mean scores for the Bully + Victim group were significantly higher than those for the Neither group for somatic complaints, anxious/depressed, social problems, thought problems, delinquent behavior, aggressive behavior, and for the internalizing and externalizing scales. The mean scores for the Victim group were significantly higher than those for the Neither group for anxious/depressed, social problems, and attention problems. For adolescent boys, mean scores for the Victim group were significantly higher than those for the Neither group for withdrawn, anxious/depressed, social problems, attention problems, and the internalizing scales. For adolescent girls, mean scores for the Victim group were significantly higher than those for the Neither group for withdrawn, somatic complaints, anxious/depressed, social problems, and the internalizing scale.	One way ANOVAs	6
Lee & Kim (2017); Korea (57) (n = 2,283)		Violent parenting associated with cyberbullying perpetration.	NR	V – depressive mood, anger, violent parenting and peer victimization, conventiona and cyber delinquent behaviour. The more the reported peer victimization, the higher the scores for anger (β = 0.095 0.095, p < 0.001) and for depressive mood (β = 0.172, p < 0.001). Children subjected to greater peer victimization presented a higher tendency to be involved in both conventional delinquent behaviors (β = 0.173, p < 0.001) and cyber delinquent behaviors (β = 0.129, p < 0.001). Third, the pathway from peer victimization to conventional delinquent behaviors, anger (β = 0.019, 95% CI [0.009, 0.030]) and depressive mood (β = −0.010, 95% CI [−0.020, −0.001]) were shown to have a significant mediating effect. Fourth, in accordance with the pathway from peer victimization to cyber delinquency, both anger (β = 0.015, 95% CI [0.012, 0.042]) and depressive mood (β = 0.011, 95% CI [.001, 0.034]) were shown to have a significant mediating effect.	SEM	6
Li et al. (2018); China (58) (n = 1,742)	Self-identification as LGB or “unsure”: After controlling for potential confounding variables, sexual identity continued to be associated with depressive symptoms. Male students who self-identified as LGB (AOR = 6.16; 95% CI = 2.13-17.83) or were “unsure” of their sexual identity (AOR = 1.66; 95% CI = 1.04-2.65) had higher odds of depressive symptoms compared with heterosexual male students. + Female students who self-identified as LGB (AOR = 2.29; 95% CI = 1.13-4.63) had higher odds of depressive symptoms compared with those who were heterosexual	NR	NR	V: LGB, Recent high academic pressure Alcohol use, Being bullied in school,Sexual abuse,Smoking Outcomes: Depressive symptoms Factors for depression: LGB: prevalence of depressive symptoms in the past 12 months was substantially higher among LGB students compared with those who were heterosexual (62.5% vs 18.7% for males, P <.001; 40.5% vs 22.2% for females, P = .005; **Table 2**). High academic pressure (P <.05 for all male and female comparisons) + male high academic pressure (AOR =1.79; 95% CI = 1.25-2.56) + female + female academic pressure (AOR =2.77; 95% CI = 1.86-4.13) Alcohol use (P <.05 for all male and female comparisons) + male alcohol use (AOR =1.69; 95% CI = 1.16-2.47) + female alcohol use (AOR =2.29; 95% CI = 1.45-3.62) Being bullied in school (P <.05 for all male and female comparisons) + male being bullied at school (AOR =2.56; 95% CI = 1.80-3.63) + female being bullied at school (AOR =1.99; 95% CI = 1.33-2.97) Sexual abuse (P <.05 for all male and female comparisons) + male sexual abuse (AOR =2.31; 95% CI = 1.16-4.59) + female sexual abuse (AOR =3.16; 95% CI = 1.23-8.13; **Table 3**). Smoking: prevalence of depressive symptoms was nearly 2 times higher among students who smoked in the last 30 days than among those who did not (35.5% vs 17.4%; P <.001) (males only) + male cigarette smoking (AOR = 2.39; 95% CI = 1.54-3.73),	LR	7
Ma et al. (2018); Taiwan (59) (n = 730)	Both support seeking strategies and problem-solving strategies buffered the effects of BV in Taiwanese adolescents from loneliness and depression	NR	NR	V – Coping strategies, Peer victimization, Psychological diress (loneliness, depression) Bivariate correlation showed that perceived peer victimization was associated with depression (r=0.40, p<0.01)	CFA with a good model fits	7
Min et al. (2015); South Korea (60) (n = 1,198)		Childhood trauma, neglect and physical abuse were associated with victimization and perpetration	NR	V - BP and BV, suicidal ideation, delinquency, history of childhood trauma, depressive symptoms Bullying victimization and perpetration are related to suicidal ideation via the mediation of depressive symptoms. Bullying victimization and perpetration were associated with depressive symptoms and suicidal ideation especially in females. Bullying victimization was associated with depressive symptoms (β=0.13, P<0.01) but the effect was significant only in females (β=0.17, P<0.001). In addition, depressive symptoms were significantly associated with suicidal ideation (β=0.47, P<0.001) in both males (β=0.39, P<0.001) and females (β=0.49, P<0.001). Bullying perpetration was directly related to suicidal ideation (β=0.13, P<0.01), independent of depressive symptoms.	SEM	5
Seo et al. (2017); South Korea (61) (n = 2,936)		Poor perceived relationship with parents associated with bullying victimization.	Lower socioeconomic status was associated with bullying victimization.	V – demographic, BV, depressive symptoms Poor academic achievement, depressive symptoms were associated with bullying victimization.	LR	5
Shao et al. (2014); China (62) (n = 2,457)	Adol children divided into 4 categories -Class 1 (16.2%) of the students. “aggressive group”. -Class 2 (9.2%) had a high response probability on aggression items and on victimization items, “aggressive victimized group.” -Class 3(47.2%) had low response probabilities on the 4 aggressive items and 4 victimization items, and this type was named as the “general group”. -Class 4 (27.4%) had lower response probabilities on the 4 aggressive items and moderate response probabilities on items 5, 6 and 8, and this category was named the “victimized group”;	NR	NR	V – campus aggression and bullying, loneliness, depression, anxiety, Peer and teacher supports, Academic achievement Aggressive victims scored the worst in questionnaires. Protective factors – peer and teachers supports had important influences on children’s aggressive and victimizaed behaviours. Compared to ‘general children’ aggressive victims, aggressive children and victimized children had low probability of receiving peer supports.	LCA – latent class analysis	7
Tang et al. (2018); China (63) (n = 1,663)	LBC had More School BV occurring moderately often (OR=1.66,95%CI 1.06-2.59*) or very often (OR=2.37,95%CI 1.43-3.93***) which was predictive of depression	NR	NR	V – school bullying, self-esteem, panic symptoms, depression, severe psychological distress (SPD), In LBC children predictors of depression; low self esteem relative to high self-esteem (OR=2.47,95%CI 1.67-3.65***), only-child status (OR=1.55,95%CI 1.14-2.09*), being cared for by a relative as opposed to mother only (OR=2.84,95%CI 1.17-6.90*)	Multinomial LR	9
Wang et al. (2019); China (64) (n = 1,347)	NR	NR	NR	Bullying was associated with depression (Multivariate OR=1.89, CI 1.12-3.18, p<0.05) in the full cohort.	Univariate LR	7
Wang et al. (2020); China (65) (n = 569)	Victimized youth who used non confrontation strategy were more prone to suffer from loneliness	NR	NR	V – peer victimization, conflict resolution strategies, depressive sympotms, loneliness Peer victimization was (+) assoc with depression (r=0.58**) and loneliness(r=0.59**). Relation b/w peer victimization and psychological problems were attenuated by solution-orientation strategy. Solution-orientated strategy was negatively correlated with depressive symptoms (r=-0.52**) and loneliness (r=-0.56**) whereas non confrontation was (+) correlated with depressive symptoms (r=0.37**) and loneliness (r=0.38**)	MANOVA – Multivariate Analysis of Variance	7
Xiong et al. (2019); China (66) (n = 194)		maternal psychological control (+) effect on depression β=0.27***)	peer BV was significantly positively correlated with depression (r=0.46, p<0.001) From hierarchal multiple regression analyses, peer BV had a significantly positive effect on depression (β=0.39***)	V – peer victimization, maternal control, self injury behaviours, depression, loneliness Maternal psychological control was (+) related to depression while maternal behavioural control was (-) related to depression. Mothers who exerted high psychological control, and high behavioural control reduced negative effect of peer BV on self-injury behaviors. (simple slope = 0.08, t=0.73 p>0.05)	multiple regression analyses,	7
Yeh et al. (2019); Taiwan (67) (n = 474)	NR	NR	NR	V – School BV, depression, anxiety, insomnia, ADHD symptoms Among children with ADHD, Verbal and relational bullying was a/w depression (β=0.265-0.301, p<0.05) Physical bullying was a/w depression (β=0.114-0.180, p<0.05)	LR	9
Yen et al. (2014a); Taiwan (68) (n = 6,406)	NR	NR	NR	V – mental health problems, alcohol abuse, inattention, hyperactivity/impulsivity Associations between bullying involvement with depression: all p<0.001 Victim (passive) beta 0.279, t 22.311 Victim (active) beta 0.182, t 14.378 Perp (passive) beta 0.183, t 14.393 Perp (active) beta 0.114, t 8.893	Multiple LR	8
Yen et al. (2014b); Taiwan (69) (n = 5,252)		NR	NR	V – BV, BP of passive and active, BMI, social phobia, depression, self-esteem, suicidality BMI was associated with bullying perpetration. Victimization of passive and active bullying and perpetration of passive bullying but not perpetration of active bullying, had a mediating effect on the relationships between increased BMI and all four mental health problems (social phobia, depression, suicidality and low self-esteem) among adolescents	Multiple Regression Analysis, SEM	9
Yen et al. (2014c); Taiwan (70) (n = 251)	Older age and traditional passive bullying victimization were associated with cyberbullying victimization. Older age, higher reward responsiveness, combined type ADHD, more severe internet addiction, and traditional passive bullying perpetration were associated with cyberbullying perpetration,	NR	Higher paternal occupational socioeconomic status associated with cyberbullying victimisation	V – CyBP, depression anxiety,suicidality	Multiple LR	7
Yin et al. (2017); China (71) (n = 755)	NR	NR	NR	V- BV, peer support, active coping, depression, stressful life events Peer victimization association with depression (β=0.28, p<0.001) Boarding female students had higher levels of peer support and active coping than males in this sample. The association between victimization and depression was stronger in boys (Beta=0.39, p<0.001) than girls (beta=0.19, p<0.001). Peer support had a direct negative effect on depression (β=−0.26, p < 0.001) and active coping for the whole group (β=−0.09, p < 0.05) but not active coping. Instead, active coping moderated the effect of peer victimization on depression for the whole group but peer support did not have this moderating effect.	SEM	8
Yun & Kim (2016); South Korea (72) (n = 1,793)	Girls (B = 0.32) were, in general, more likely than boys to feel depressed (B = 0.32 p<0.001). Sociodemographic: Age did not exhibit significant effects.	In BV adol had significantly lower levels of depression with higher attachment to their mothers and fathers than those who had a lower attachment (i.e Interaction effects of parental attachment and bully/victim status predicting depression – (Maternal attachment x Bully-victim B = -0.40* SE 0.16 β=-0.31 R^2^ = 0.21; Paternal attachment x Bully-victim B=-0.38* SE 0.15 β=-0.28) (β = - 0.33, *p* < 0.001)	NR	V – BV,BP,slef injury, suicidality, maternal and paternal attachement, demographic The higher the SES (B = -0.22, p<0.001), the lower the level of depression felt by respondents. #respondents who have been involved in bullying either as a perpetrator or a victim both report significantly greater levels of depression it appears that bully-victims (B = 1.20) experienced the highest level of depression anxiety, followed by bullies only (0.90) and, lastly, by victims only (0.63). (p <0.01).	OLS regression model, LR	9
Zhou et al. (2017); China (73) (n = 448)	NR	NR	NR	V – BV,BP, depression, resilience, mindfulness Bullying victimization was positively correlated with depression, and negatively correlated with resilience and mindfulness (p < 0.001) As can be seen from the mediator and dependent variable model, after controlling for gender and grade, bullying victimization negatively predicted resilience (β = − 0.22, p < 0.001), resilience negatively predicted depression (β = − 0.32, p < 0.001), and bullying victimization positively predicted depression (β = 0.14, p < 0.01). Besides, the interaction of bullying victimization and mindfulness had a significant effect on depression `, and the interaction of bullying victimization and mindfulness had a significant effect on resilience (β = 0.23, p < 0.001). These results indicated both the relation between bullying victimization and depression and the relation between bullying victimization and resilience were moderated by mindfulness	Linear regression	7
Pan & Spittal (2013); China (74) (n = 8,182)	NR	NR	Prevalence of racial bullying in Urumqi (2.08%) was significantly higher than that in Beijing (0.72%) and Wuhan. (0.67%).	V - racial and religious bullying, health-related outcomes, age, sex, parents education, hunger (control variables) Religious bullying was significantly assoc with depressive symptoms among males (AOR 8.85 95%CI 1.96-40.02**) and females (AOR 11.50 95% CI 1.62-81.42*) In females racial bullying was associated with depressive symptoms (AOR 2.19 95%CI 1.04-4.61)	Chi-square tests, GLIMMIX procedure	9
Zhao et al. (2021); China (75) (n = 16,380)	Sexual Minority youths(n=1360) were more likely to experience maltreatment (AOR range: 1.25-2.46**) and BV (AOR range: 1.38-1.77**)m and a series of health problems (AOR range 1.85-3.69***) (except for physical abuse victimization in boys) than youths with opposite-sex attraction (n=15,020)	NR		V – sexual minority, heterosexuals, childhood maltreatment (physical,emotional, sexual abuse) TBV, CyBV BV could partially explain the association between sexual minority status and psychological distress (β=0.040***SE0.011, 95%CI 0.02,0.064 for boys; (β=0.031***SE0.006, 95%CI 0.021,0.047) for girls	Multivariate LR, SEM	8
Mei et al. (2021); China (76) (n = 2,956)	Social Anxiety correlated positively with bullying victimization (r=0.121**). Sleep duration had a mediating effect through negative association with social anxiety (r= -0.081**)	NR	NR	V – BV, social anxiety, depression, sleep duration	Pearson correlation, SEM	9
Lai et al. (2023); China (77) (n = 19,809)	Adolescents with negative coping style (n=10,006) who experience BV i.e relational victimization (β=3.42*** 95%CI 3.07,3.77) and cyBV(β=4.82*** 95%CI 4.25,5.39) were more likely to have anxiety than those with positive coping style Adolescents with negative coping style (n=10,006) who experience BV i.e relational victimization (β=7.94*** 95%CI 7.22,8.67) and cyBV(β=10.32*** 95%CI 9.13,11.51) were more likely to have depression than those with positive coping style	NR	NR	V – BV, coping style, anxiety symptoms, depressive symptoms,	Univariate Linear mixed effects models	9
Liu et al. (2020); China (78) (n = 8,918)		Sibling BP (n=1230) (verbal, physical or relational) had a higher risk of major depression (AOR=1.44 95%CI 1.26 to 1.64***) and anxiety (AOR=1.63 95%CI 1.42 to 1.87***) than those not involved with sibling BV Sibling BV(n=1235) (verbal, physical or relational) had a higher risk of major depression (AOR=1.49 95%CI 1.32 to 1.68***) and anxiety (AOR=1.68 95%CI 1.48 to 1.90***) than those not involved with sibling BV		V- anxiety, depression, sibling bullying, peer bullying, confounding demographics	LR	7
Guo et al. (2022); China (79) (n = 3,635)	Aggressive behavior as a maladaptive reaction is a mediating factor	Perceived social support from parents and other relatives is a vital protective factor	Perceived social support from friends is a vital protective factor	V – BV, aggression, perceived social support, mental health measures – anxiety, depression, subjective well being	Exploratory factor analysis	5
Peng et al. (2022); China (80) (n = 3,062	Sexual orientation was significantly associated with bullying victimization. LGBTQ adolescent have higher rates of bullying from peers, siblings and both.	LGBTQ adolescents (n=668) had higher odds of experiencing sibling victimization only (OR=1.41* 95% CI 1.08, 1.85), or peer victimization only (OR=1.54** 95%CI 1.13, 2.08) or both sibling and peer victimization (OR=2.23*** 95%CI 1.48, 3.34) compared to Heterosexual adolescents (n=2394)	NR	V – depression, anxiety, sibling and peer BV, sexual orientation, demographic	Multinomial LR	8
Zhu et al. (2020); China (81) (n = 18,452)	Adolescence with repeated victimization (more than once in the preceding year)(n=8167) have significantly higher levels of depression (Contrast 5.33* t=32.94 β=0.29, lower self-esteem (Contrast -0.91* t=-11.91 β=-0.11, and poorer overall health (Contrast -0.462* t=-35.06 β=-0.30, when compared with one-time victimization (n=5082) or those with none(n=5203)	NR	NR	V – child victimization, self-esteem, depression, Health status	MANOVA	7
Wen et al; China (2022) (82) (n = 1,481)		NR	NR	V – demographic, BP,BV, anxiety, depression, Adolescents with no or mild depression had significantly lower bullying perpetration than those with moderate-to-severe depression. Those with no or mild anxiety had higher perpetration than those with moderate-to-severe anxiety.	Multivariate analysis (MANCOVA)	4
Zhu et al. (2021); China (83) (n = 3,232)	Males more likely to report cyberbullying victimization in preceding year than females (n=122 vs 75 ***) (β=3.74***; β=4.48***) and PTSD (β=7.16***; β=4.77***) on (OLS) ordinary least squares regression	Parent-child attachment significantly moderated effects of cyBV on depressive and PTSD symptoms in lifetime and preceding year cyBV. In adol.with greater levels of parent-child attachment, the lifetime and preceeding year cyBV had less effects on adolescents depressive symptoms (β_lifetime_= -0.33***; β_past year_ = -0.57***) and PTSD (β_lifetime_= -0.40**; β_past year_ = -0.54*) compared with those with lower attachment scores	NR	V -CyBV, parent child attachment, Health, depressive symptoms, PTSD, Substances misuse and gambling, socio demographic Adolescents with Lifetime (n=710) and preceding year (n=202) cyBV were more likely to engage in problem drinking (OR=1.64***; OR=1.84**) cigarette smoking(OR=1.69***; OR= 2.21***)and gambling(OR=1.35*; OR=1.97**) compared to non-victimized peers.(n=2491_Lifetime_ to 2991_past yr_) Lifetime and preceding year cyberbullying victimization reported significantly lower levels of overall health (β= -1.58***; β= -2.22***) and more severe depressive symptoms. (β=3.74***; β=4.48***) and PTSD (β=7.16***; β=4.77***)	(OLS) ordinary least squares, multiple regression analysis	8
Cao et al. (2021); China (84) (n = 2,022)	Bullying victimization effect on depression is in part mediated by internet addiction and sleep quality BV→IA→ SQ→ DS (B=0.023, SE 0.004, (LL,UL 0.016,0.032) BV, internet addiction, sleep quality and depression were positively correlated**	Students with high BV scores were associated with males (n=1009; M=6.57, t or F=6.27***), lower household socioeconomic status (n=214; M=6.67, t or F=6.60**), not living with parents (n=124; M=6.65, t or F=3.01*), tobacco use (n=122; M=6.94, t or F=3.14**), and alcohol use(n=471; M=6.69, t or F=4.95***),	NR	V - BV, depression, internet addiction, sleep quality	Pearson Correlation, mediation model	9
Liu et al. (2023); China (85) (n = 3,841)	Homosexuality (n=74) (AOR 6.398* 95%CI 3.321 to 12.325), bisexuality (n=139) (AOR 3.146*; 95%CI 1.499 to 6.603) and uncertainty of sexual orientation(n=588) (AOR 2.341*; 95% CI 1.516 to 3.615) were significantly associated with a combination of traditional and cyBV.	NR	NR	V – BV, depressive mood, anxiety, sexual orientation, sociodemographic #Sexual minority students, esp bisexual students have a higher risk of depressive mood (AOR 2.349*; 95% CI 1.664 to 3.316) and anxiety mood (AOR 3.049*; 95% CI 2.150 to 4.324).	Multivariate LR	9
Fan et al. (2021); China (86) (n = 1,174)	Sense of security partially mediated the correlation of victimization with adolescent depressive symptoms. Victimization (N=1174) was negatively associated with sense of security (β=-0.31, SE=0.03, t=-11.07***) and sense of security was negatively associated with depressive symptoms (β=-0.48, SE=0.02, t=-19.41***) and the direct effect was 0.15 (95% CI 0.12,0.18) accounting for 41.7% of the total effect.	NR	NR	V- sense of security, BV, depression, psychological capital	Correlation, SEM	9

NR, Not reported; BV, bullying victimization; BP, bullying perpetration; B-V, bully-victims (subgroup who are victimize and bully); cyBV, cyberbullying victimization; cyBP, cyberbullying perpetration; TBV, Traditional Bullying victimization; Adol, adolescents; *p<0.05 **p<0.01 *** p<0.001; V, variables; CoV, covariates; T1,T2, T3, Time 1, Time 2; β, Beta coefficient; B, unstandardized Beta coefficient; AOR, adjusted odds ratio; OR, odds ratio; CI, confidence interval; Assoc, associated; M, Mean; SD, standard deviation; SE, standard error; LR, Logistic Regression; (+) positive; (-) negative; LLCI, Lower limit confidence interval 95%; ULCI, Upper limit confidence interval 95%; SEM, Structural Equation modelling; PTSD, Post-traumatic stress disorder; QOL, Quality of life; BAS, behavioral approach system; BIS, behavioral inhibition system; BMI, Body mass index; ADHD, Attention-deficit/hyperactivity disorder; ASD, Autism spectrum disorder; LGB, lesbian, gay, or bisexual; LBC, Left behind children; LGBTQ, Lesbian, gay, bisexual, transgender, and queer.

#### 3.3.1 Individual factors

##### 3.3.1.1 Personal traits/style

The findings of 3 studies with good NOS scores with larger sampler sizes (77, 81, 86) were examined in more detail. Adolescents with a high sense of security and positive coping style were more resilient to the negative effects of victimization. Adolescents with low self-esteem were more susceptible to bullying victimization and its negative health effects.

*Self-esteem –* Adolescents with repeated victimization had significantly lower levels of self-esteem and overall health, compared with none or one-time victimization (81). In LBC, depression was higher in children with low self-esteem compared to those with high-self-esteem (odds ratio (OR) 2.47***) (63). Active or passive victimization also had mediation effect on the relationship between increased BMI and low self-esteem (69). In smaller studies, low self-esteem predicted cyberbullying victimization (25), while friendship intimacy correlated positively with self-esteem (29).

*Sense of security* is a perception and reaction of one’s security state apart from anxiety and fear (Maslow’s hierarchical theory of needs) (86). Sense of security partially mediated depression risk, with more secure individuals experiencing less depression. Victimization (N = 1,174) was negatively associated with sense of security, and sense of security was negatively associated with depressive symptoms (86).

*Coping style* is an approach where individuals can use their cognition and strategies to manage stressful events. They are predominantly positive (problem solving, seeking support) or negative (avoiding, enduring) (77). Adolescents with negative coping styles (n = 10,006) who experienced relational victimization (β = 3.42***) and cyberbullying victimization (β = 4.82***) were more likely to have anxiety than those with a positive coping style. They were also more likely to have depression than those with a positive coping style (β = 10.32***) (77).

In other smaller studies, ‘active coping’ (71), high support-seeking (59), mindfulness (73), self-compassion (49), and ‘solution-oriented’ conflict resolution skills (65), resulted in better outcomes from victimization. Individuals with high interdependence (22) and hopelessness (49) had poorer outcomes.

*Mindfulness* (which refers to a trait of being aware of ongoing physical, cognitive and psychological experiences, and requires attention control, self-awareness and self-empathy or acceptance) moderated both bullying victimization on resilience (β = 0.23***) and bullying victimization on depression (β = − 0.11**), and this was seen more in children with low mindfulness (73).

*Self-compassion* moderated the effects of victimization on depression (47). Self-compassion is described as a ‘kind and understanding disposition exhibited towards the self in times of trouble and failures’ (32, 47). Self-compassion negatively predicted depression in bullying victimization (32, 47). In individuals with low self-compassion, cyberbullying victimization was associated with hopelessness (β = 0.70***) and depression (β = 0.36*).

*‘Interdependence’* is a construct where individuals rely on emotional connection with others for their self-view. Studies by Kawabata et al. (n = 387) (22) and Yin et al. (71) found that adolescents who placed great value and emphasis on relationships (i.e., highly interdependent) were more likely to have subsequent depressive symptoms if they experienced relational victimization) (*r* = 0.72***) (22).

##### 3.3.1.2 Age

In 2 large studies with good NOS scores, the effects of age were opposite (31, 43). Chan et al. (43) (n = 18,341) found age was negatively associated with bully victimization (adjusted odds ratio (AOR) 0.91***). Li et al. (31) (n = 10,279) on the other hand, found bullying victimization was higher in older adolescents (n = 2,498) (**Table 5**). Bullying victimization increased with increasing age in three other studies (49, 51, 70), with a higher risk of depression in older adolescence (49, 51). Age was a moderator of bullying victimization and depression in older females through sleep problems (23). In only one study (n = 661) in Chinese adolescents did bullying victimization lead to higher rates of depression in younger children (25) where students in Grade 7 had greater symptoms of depression (*F*_(1,594)_ = 4.13*) than in Grade 8.

##### 3.3.1.3 Gender

In 3 studies with good NOS scores and larger sample size (28, 44, 45), male adolescents were more likely than females to be involved with traditional and cyberbullying victimization/perpetration (**Table 5**). In 3 other good quality studies, sexual minority status was overwhelmingly associated with victimization and depression risk (75, 80, 85).

Chinese boys (n = 1,019) scored higher at T_1_and T_2_ than girls (n = 668) on bully victimization (26) (**Table 5**). Male gender (n = 901) was also associated with cyberbullying victimization (OR 1.62) and cyberbullying perpetration (OR 1.59) more than female gender in Taiwan (42). Taiwanese males were also more likely to be cyberbully perpetrators (OR 1.57) and cyberbully-victims (OR 3.17) than females (43).

Sexual minority status was strongly associated with bullying victimization (74, 79, 84) and depression risk. In Liu et al. (85), homosexuality (n = 74) (AOR 6.40*), bisexuality (n = 139) (AOR 3.15*) and uncertainty of sexual orientation (n = 588) (AOR 2.34*) were significantly associated with a combination of traditional and cyberbullying victimization when compared with heterosexual status. Sexual minority students, especially bisexual students, had a higher risk of depressive (AOR 2.35*) and anxious mood (AOR 3.05*) compared to heterosexual students. In a large Chinese study (75) (n = 16,380), sexual minority youths (n = 1,360) were more likely to experience maltreatment (AOR range: 1.25-2.46**) and bully victimization (AOR range: 1.38 – 1.77**) and a series of health problems (AOR range: 1.85 – 3.69***) than youths with opposite-sex attraction. In Peng et al. (80), LGBTQ adolescents (n = 668) had higher odds of experiencing sibling victimization only (OR 1.41*), or peer victimization only (OR 1.54**) or both sibling and peer victimization (OR 2.23***) compared to heterosexual adolescents (n = 2,394).

##### 3.3.1.4 Other factors

*Cognitive Ability and Academic Achievement* – Victimization scores were higher for those with low cognitive ability scores (n = 2,631) (M = 11.6 vs. 10.4***) when compared to adolescents with highest ability scores (n = 2,210) (31). In the same study (n = 10,279), victimization was also associated with poorer academic achievement at 2 and 5 year follow up (β_2yr_ = -0.04^**^, β_5yr_ = -0.03*).

There were 2 good quality studies on BMI (26, 69). Overweight adolescents (n = 5,252) had increased risk of victimization and poorer mental health outcomes (69). Increased BMI was positively associated with severity of victimization (active and passive bullying, perpetration of passive bullying) and these severities were positively associated with severities of social phobia, depression, suicidality and low self-esteem (**Table 5**). The association between BMI and depressive symptoms was also significantly mediated by peer victimization and sleep problems in Chang (n = 1,893) (26). Higher BMI predicted more peer victimization, leading to more sleep problems, and to higher levels of depressive symptoms (Total effect β = -0.039, SE 0.020, 95% CI: -0.079 to 0.001).

#### 3.3.2 Family factors

*Parent – child attachment* was examined in 3 good quality papers with larger sample size (44, 72, 83). In Chang et al. (44) (n = 1,867), adolescents with cyberbullying victimization had lower parental attachment (OR 0.79) compared to those without cyberbullying victimization. Parent – child attachment significantly moderated effects of cyberbullying victimization on depression in a study by Zhu et al. (n = 3,232) (83). In adolescents with greater levels of parent – child attachment, the lifetime and preceding year cyberbullying victimization had less effects on adolescents’ depressive symptoms (β_lifetime_ = -0.33***; β_past year_ = -0.57***) and PTSD (β_lifetime_ = -0.40**; β_past year_ = -0.54*) compared with those with lower attachment scores (83). Parent – child attachment also moderated the effects of victimization on depression in all three groups (bully, bully-victim, victim) in a Korean study of 1793 adolescents (70). In adolescents experiencing victimization, significantly lower levels of depression were reported by those who held a higher attachment to their parents than those who had a lower attachment (β = - 0.33***) (83).

*Family dysfunction* was explored in 3 good quality papers with larger sample size (31, 47, 52) and was associated with increased bullying victimization, perpetration and depression. In Chen et al. (n = 18,341) (47), parental divorce/separation/parent widow status (AOR 1.37 – 1.68*) was associated with bully perpetration. All types of family victimization (conflicts within family) were associated with greater risk of bully victimization (AOR 1.99 – 5.36***). All types of family victimization (except neglect) were associated with cyberbullying victimization (AOR 2.24 – 5.36***) (47). In a Korean study of 10,453 adolescents (52), high levels of parental abuse (β = 0.05***); high levels of parental neglect (B=0.025*); and high levels of family dysfunction (β = 0.053**) were associated with direct cyberbullying victimization (adjusted R^2^ = 0.173). In Li et al. (n = 10,279) (31) traditional bullying victimization scores were higher for adolescents who were not close to parents (n = 5,335) (M: 11.3 (4.4) vs. (10.6 (4.2) ***) in adolescents who were closer to parents; and for adolescents not living with parents (n = 3,084) (M: 11.3(4.4) vs. 10.6(4.2) ***) compared to those living with parents. Cyberbullying victimization was also associated with fewer parental restrictions (OR 0.89) than in children with more parental oversight in the Taiwan study (n = 1,867) (44).

*Sibling association with bullying victimization* was examined in four large studies (31, 43, 78, 80). Liu et al. (n = 8,918) (78) specifically explored the sub-types of sibling bullying among Chinese children and adolescents. Sibling bullying perpetration (n = 1230) (verbal, physical or relational) had a higher risk of major depression (AOR 1.44***) and anxiety (AOR 1.63 ***) than those not involved with bullying. Sibling bullying victimization (n = 1,235) (verbal, physical or relational) had a higher risk of major depression (AOR 1.49***) and anxiety (AOR 1.68***) than those not involved with sibling victimization. Peng et al. (80) found that sexual minority adolescents (i.e., LGBTQ) experienced more bullying from their siblings. Adolescents with siblings were more like to be involved in bullying victimization and perpetration. In Chen et al. (n = 18,341) (43), having siblings (AOR 1.36* – 1.41***) was associated with bullying perpetration and bullying victimization (AOR 2.00***) compared to adolescents with no siblings. In Li et al. (n = 10,279) (31), traditional bullying victimization scores were also higher for those with siblings (n = 6,774) (M: 11 vs. 10.5***), compared to those without.

#### 3.3.3 Community factors

##### 3.3.3.1 Peer relationships

*Friendship intimacy* (29, 37, 41, 62, 71) strongly contributed to reduced risk of depression, improved self-esteem and well-being, with less bullying victimization. Two good quality papers explored this theme (37, 41). In Yang et al. (37), (n = 2,339), adolescents who had resilient profiles [n = 188 (8%)] had higher levels of bullying victimization but low levels of depression and high levels of subjective well-being. Higher level of friend support contributed to a ‘resilient’ profile. Adolescents in the ‘Resilient’ profile group benefitted from more teacher and peer support. Support from teachers and peers correlated positively with subjective well-being *r* = 0.28***; *r* = 0.31***); correlated negatively with bullying victimization (*r* = -0.20***; *r* = -.0.51***); and correlated negatively with depressive symptoms (*r* = -0.23***; *r* = -0.30***) (35). Higher friendship quality also dramatically increased odds of moving from a victim or bully-victim to non-involved adolescent [Victim to Uninvolved (OR 7.31***)]; [Bully-victim to Uninvolved (OR 17.07***) (41). In a smaller study with high NOS score, greater friendship intimacy (n = 450 in Yang et al.) was measured by adolescents’ level of intimacy with up to four best friends. T_1_ Bullying victimization and T_1_ Friendship intimacy was a moderator and correlated positively with T_2_ self-esteem (β = 0.090*).

Conversely, adolescents with lower levels of peer support (β = -1.05**, OR 0.35) were more likely to be classified as ‘Adverse’ profiles, rather than the ‘Resilient’ profile in Yang et al. (37). Adverse profiles [n = 89 (3.8%)] had the highest level of bullying victimization, highest levels of depression, and lowest levels of subjective well-being.

##### 3.3.3.2 School Environment

Adolescents who have been bullied have less depression if they have more *teacher support* (35, 60), better *peer support* (50, 71) and higher levels of *school connectedness* (the belief that others in school care about their learning, and them as individuals) (51) (**Table 5**).

In contrast, adolescents with lower levels of teacher support (β = -0.87**, OR 0.42) were more likely to be classified as ‘Adverse’ profiles, rather than the ‘Resilient’ profile in Yang et al. (37). Bullying by teachers (46, 51) (including quarrelling with teacher, emotional or physical punishment by teacher) correlated with depression. Surprisingly, adolescents within low victimization environments had more reactive aggression, and victims of physical bullying had more depression compared to those in higher victimization environments. In Zhao et al. (n = 691) (42), physically and relationally (but not verbally) victimized adolescents in healthy cliques with lower victimization norms reported committing more reactive (not proactive) forms of aggression ((Externalizing)B_physicalV_ = -0.10*; B_relationalV_ = 0.10*) and having more depressive symptoms 2 years later ((Internalizing) B_physicalV_ =0.20**) (**Table 5**). This may be explained by the ‘healthy context paradox’ which describes children in cliques with lower victimization levels reacting more aggressively as there is greater disparity with the majority of peers (i.e., social misfit), further aggravating peer isolation. LBC also experienced more severe bullying in schools (OR 2.37***) (63).

### 3.4 Social determinants of health

Higher parental education was protective (29) through higher paternal occupational socio-economic status, but the risk of cyberbullying victimization also increased (70), possibly due to increased online access. Lower socio-economic status (25, 31, 61), lower maternal education (31, 47) and paternal unemployment (31, 47) were associated with higher rates of bullying victimization (47, 61) and depression (25).

### 3.5 Type of bullying with higher risk of depression

Higher depression levels or poorer well-being resulted from bullying perpetration of passive and active bullying (68), higher levels of bullying victimization (37), bullying by being threatened or intimidated (33), poly-victims (those experiencing more than one type of bullying (43, 81), and those in the bully-victim group (56). In a Japanese study (56) of 486 adolescents, boys who are bully-victims (n = 35) had significantly higher mean scores when compared with the neither group (n = 146) in terms of social problems (M=5.06***), attention problems (M=7.29 ***), aggressive behaviour, (M=9.80***), and the externalizing scale (M=12.23***) (**Table 5**). For adolescent girls, the mean scores for the bully-victim group were significantly higher than those for the Neither group for all the dimensions and subscales measured above.

### 3.6 Vulnerable Groups

#### 3.6.1 Left behind children

LBC accounting for around 69 million children in China are children left in the care of grandparents or one parent in rural areas when their parent(s) move to cities in China for work (61, 63, 64). LBC have a higher risk of bullying victimization and being depressed as a result (61). The presence of high maternal psychological control increased bully victimization in LBC whereas a with the presence of both maternal high psychological and behavioural control on children, the negative effect of bully victimization on self-injury was buffered. When left behind women had low psychological control, then higher maternal behaviour control worsened the negative effect of peer victimization on self-injury (64). In LBC, depression from bullying victimization also increased with being an only child (OR 1.55***), low self-esteem (OR 2.47***), being in care of another relative as opposed to mother only (OR 2.84***) (63).

#### 3.6.2 Ethnocultural bullying

Ethnocultural (i.e., racial or religious) bullying was significantly associated with depression. Racial bullying was associated with depression in females (AOR 2.19*) and religious bullying was associated with depression in males (AOR 8.85**) (74).

#### 3.6.3 Adolescents with developmental conditions and existing mental health issues

Two studies (54, 70) showed that children with developmental disorders such as ADHD had depressive symptoms associated with bully victimization (β = 0.190**) or perpetration (β = 0.228***).

Adolescents with prior mental health conditions had higher risk of both being victims and perpetrators (36). Those with existing depression were more likely to be victims or bully/victims (27, 41), or bully perpetrators (36, 82) and those with social anxiety (76) were more likely to be victims. Adolescents with mild anxiety were more likely bully perpetrators than those with more severe anxiety (82).

*Problematic Internet Use* – Korean boys had a higher prevalence of PIU compared to girls (16.1% vs. 8.1%) (55). All groups including victim only (OR 2.36***), bully only (OR 1.66*) and bully-victim group (OR 2.38***) had a higher likelihood of PIU than the group who were neither victims nor bullies. Cyberbullying victims were also more likely to be depressed (OR 4.2 ***) in this study (55). In a Taiwan study of 1808 high school students, internet addiction was also found to be associated with depression (OR 1.92) (44).

## 4 Discussion

### 4.1 Prevalence rates of bullying victimization

In East Asian countries, the prevalence rates of bullying and victimization in adolescence varied greatly and ranged from 6.1 – 61.3% in traditional bullying victimization, and 3.35 – 74.6% in cyberbullying victimization. This variation in prevalence rates of bullying victimization is also observed globally. Data from the Global School-based Student Health Survey GSHS (2003 – 2015) of school children aged 12 – 17 years showed that the Eastern Mediterranean Regions (including the middle East) had the highest prevalence (45.1%) (87) compared with rates of 24% from China. Prevalence rates from Europe and North America were not covered in this study but in other studies were reported to be up to 36% of European adolescents (88) and 20.2% of American youth (89).

The possible downward trend of rates of bullying victimization in studies conducted after 2012 may reflect the global outcry of bullying in schools, raising awareness at the community level and successful national responses with the implementation of policies to prevent bullying and deal with perpetrators in schools (90).

There was also variation in prevalence rates within the three subgroups of bullying victimization (bully, victim, bully-victim) described. The prevalence rates of bully-victims in adolescents was highest in Japan (15.9%) (56) and lowest in Fujian, China (3%) (53). The literature is clear that bully-victims have the poorest functioning with poorer emotional adjustment, peer relationships and health, and longer-term outcomes including depression, suicidal ideation, substance abuse and delinquency (91, 92) compared to bullies and victims.

Bully-victims had the most dysfunction (93) and consequent poor school engagement and academic functioning. Bully-victims experienced intense emotional distress from feelings of helplessness and anxiety when victimised, to feelings of anger and frustration when bullying others. The cycle of being victimised and bullying others made it difficult for them to form positive relationships with others. Hence, they were the most socially isolated, perceived as social outcasts by peers and tended to provoke negative reactions (93). They were also more likely to experience violence and extreme discipline at home and have a chaotic family life (94). With a paucity of peer and home supports (95), they were at much higher risk of poorer mental and physical health outcomes.

### 4.2 Discussion of bullying and depression

Similar to Western cultures (96–98) there is a strong positive correlation between bullying and depression seen in East Asian adolescents. In a recent meta-analysis, the risk of depression in children and adolescents was 2.77 times higher if they were bullied (97). The aims of this scoping review are to determine if there were unique aspects of East Asian ‘collectivistic’ culture which would predispose adolescents to bullying and depression when bullying occurred, and the presence of protective factors.

The main findings are that risk and protective factors for bullying and victimization in East Asian cultures are very similar to those reported in Western cultures. The evidence being that strong relationships within families, peers and the school community coupled with adolescents’ positive coping style and view of the world are protective against the negative effects of bullying. Conversely, poor parent-child attachment amidst family dysfunction, poor engagement with peers and the school community together with a negative coping style predispose East Asian adolescents to depressive symptoms as a result of bullying victimization.

Healthy and supportive relationships within family systems, peer interactions and teacher engagement are key to increasing ‘resilience’ in East Asian adolescents who experience victimization, and this is no different to that seen in Western cultures. Studies with high NOS scores of 7 and above with larger sample sizes (above 1000) support this conclusion (**Table 5**).

#### 4.2.1 Family factors

Within family systems, poorer parent-child attachment (44, 73) or family dysfunction with separation, conflicts, abuse and neglect (31, 47, 52) were found to be significantly associated with bully victimization and risk of depression.

It is not surprising that adolescents with poorer attachment to parents are at higher risk of bully victimization and subsequent depression. Secure attachment to a caregiver from infancy provides the foundation for children to learn to trust and grow in social emotional competencies (99). These include the ability to manage difficult emotions such as fear, anger or anxiety. Insecurely attached children may become more avoidant or anxious. In the absence of secure attachment to a parent, children are likely to have difficulties regulating their emotions and as they mature, managing negative experiences like victimization (100). Adolescents with poor attachment are more likely to have low self-esteem and negative coping strategies to stress (101). They may have more emotional dysregulation with increased aggression or passivity. This further impairs their social functioning increasing social isolation, spiralling into more withdrawal, victimization and depression (102).

Similarly, adolescents from dysfunctional homes (31, 47, 52) experienced more victimization and a higher risk of depression. Parent separation/divorce is associated with bully perpetration (47). The emotional turmoil from family dysfunction, may lead to them trying to regain control by acting out these feelings. They are also susceptible to risk-taking behaviours (especially in single-father families) and vulnerable to victimization (103), probably due to lack of home supervision and susceptibility to peer pressure.

All types of family victimization were associated with greater risk of bully victimization as adolescents (47). Children who experience victimization at home are more likely to be exposed to negative parenting behaviours which may be harsh, maladaptive or neglectful (104) and model these behaviours on peers.

The finding of a sibling as bullies is concerning. Sibling bullying is common (105, 106), and mostly harmless (107). However, sibling bullying can increase likelihood of depression and anxiety. Liu et al. (78) found significantly higher risks of depression and anxiety in sibling perpetration and victimization. LGBTQ adolescents were also more likely to be bullied by siblings than heterosexual adolescents (80). Sibling victimization may be a reflection of family dysfunction and conflict while LGBTQ sibling victimization may instead be due to social stigma and internalised homophobia (108).

Two other Chinese studies showed that adolescents with siblings were more likely to be victims (31, 47) or perpetrators (43) but not necessarily of their siblings. It is possible that with larger families in rural China (31), there may be fewer resources (i.e., lower socio-economic status) (24, 31, 61), to reduce stress in the home compared to smaller families.

#### 4.2.2 Community factors

In the area of peer relationships, friendship quality and intimacy (29, 37, 41, 62, 71) strongly contributed to reduced risk of depression, improved self-esteem and well-being with less bully victimization. Higher levels of friend support, intimacy with friends and quality of friendships were protective against victimization and subsequent depressive symptoms. High quality friendship was also shown to increase the likelihood of moving from a victim or bully-victim to an uninvolved adolescent (41). This is a very hopeful finding given that bully-victims have the worst outcomes of all the bully subgroups.

Friendship support, especially high-quality friendships, in both East Asian (29, 37) and Western cultures (109), is one of the most effective protective factors against bully victimization, perpetration and development of depression in adolescents. One of the most important tasks of adolescence is to form and sustain friendships. Friendships provide emotional support and validation, buffer against isolation and loneliness, problem-solving strategies for both victims and perpetrators. Friends can also model more positive coping strategies and defend victims against bullies (110).

In the school community, adolescents with more teacher support (37, 62), better peer support and higher levels of school connectedness (the belief that others in school care about their learning, and them as individuals) (51) experienced less depression when victimized. Nurturing teachers provide students with safety from bullying, social and emotional support, and a sense of belonging (111). Adolescents who are more connected to school participate more positively with their teachers and peers and have improved academic engagement. Nurturing teachers also provide positive role models and can teach victimized adolescent more positive coping strategies in the presence of bullying victimization. This is critical at a time when the adolescent is developing their identity, sense of self and purpose. More importantly, as persons in authority teachers can stop bullying victimization when it occurs (112).

#### 4.2.3 Individual factors

Adolescents with a high sense of security, high self-esteem (63) and positive coping style (30, 77, 86) were found to be more resilient to the negative effects of bullying victimization. This was seen in both Western and East Asian cultures (30, 77, 86, 113).

A positive coping style (77) was found to reduce the risk of anxiety and depression (71) from victimization. Coping styles included problem focused/solution orientated (71), social support seeking (51), positive self-talk, emotion-focused coping (i.e., mindfulness (73) and relaxation approaches), cognitive reappraisal (71) and self-compassion (34, 49). Coping styles are potential areas for intervention where victimized adolescents can learn more positive ways to manage being bullied. For instance, adolescents who are victimized can learn how to seek social support from family, peers or teachers.

Self-esteem was an important mediator in the link between bullying victimization and depression, with low self-esteem being a risk factor for depression while higher self-esteem contributed towards resilience (114).

Sense of security was important in adolescents’ sense of self (115). When adolescents felt emotionally and physically secure they were less likely to be targeted by bullies. Secure adolescents were also more confident and assertive and sought out positive relationships. They were also more likely to seek help when victimized.

#### 4.2.4 Other findings

In both cultures, bully-victims and poly-victims fared the worst and had the highest risk of depression as a result of bullying victimization. Patterns of bullying (with gender differences) were also noted to be similar between East Asian and Western cultures. In East Asian cultures, boys were more likely to be physically bullied while girls were more likely to experience relational or verbal victimization (72). These patterns were similarly noted in Western countries (91, 116, 117). Problematic internet use (PIU) was also found to be associated with bully victimization (55) and depression (44) in both East Asian (118, 119) and Western cultures (120).

#### 4.2.5 The uniqueness in East Asian Confucian-informed cultures

‘*Being different’* – Appears to be a considerable risk factor in collectivistic cultures which promote group harmony and blending in (121). Adolescents who are in the sexual minority, LBC, have racial or religious differences, or body weight differences are vulnerable to bullying as they stand out. Of these, sexual minority status has significant implications for East Asian culture.

*Sexual minority status* (i.e., LGBTQ, bisexual status) in adolescents was found to be strongly associated with bullying victimization (75, 80, 85) and depression risk. This may be because of the strong emphasis of traditional gender roles in East Asian Confucian informed cultures. Traditional views of gender mean that any deviation from this is seen as aberrant and increases the risk of victimization. Family expectations and the concept of filial piety is the expectation that children will fulfil traditional roles of marriage, and have children to carry on the family line (122). Sexual minority status also may bring shame to families and disrupt social harmony where the needs of the group have to be placed above the needs of the individual (123). As such, sexual minority adolescents are susceptible to victimization and may have difficulties accessing support when social norms do not support LGBTQ individuals.

*Left behind children (LBC)* – LBC are at risk for bullying victimization. Although parent migration for work is not unique to China, these children are particularly vulnerable and get ‘left behind’ in developmental, learning and mental health outcomes (124). This may also account for children living in rural areas in China experiencing higher rates of bully victimization.

*Teachers as bullies* – In collectivistic cultures where group harmony is valued over the needs of an individual, adolescents are particularly susceptible to bullying from figures in authority. The finding of ‘teachers’ as bullies was indeed startling (46, 51, 52). The Confucian ethos which pervades all aspects of life in East Asian cultures may also mean that school systems may be more authoritarian and punitive rather than consultative (125). Teacher violence was prevalent, with up to 50% and 62% of children reporting corporal punishment by teachers in China and South Korea, respectively (125). Teachers’ bullying students is a much rarer occurrence in Western cultures (1.2%) (126) where the reverse occurs, with studies showing teachers experiencing bullying by students (80% of Australian teachers were bullied by students in a Latrobe study) (127). Apart from teachers, older students in schools may perpetrate acts of bullying. In East Asian countries, school environments and activities may be structured in accordance with beliefs and values to reinforce the peer group and ‘authority figures’ as the administrator of approval or rejections of behaviour (127). In a qualitative study of 41 adolescents aged 12 – 16 years, key themes such as a lack of education about bullying, poor classroom and failure of teachers to recognise and address bullying were identified (128).

*Differences in coping styles adopted* - While positive coping styles described earlier are seen in both Eastern and Western cultures, there are also differences in the preferential use of coping style in bullying victimization (129). In Western cultures where individual rights and personal autonomy are emphasized, coping styles tend to be more problem-focused, where adolescents may confront the bully or take active steps to stop the bullying. They are also more likely to talk about their emotions and experiences through counselling. In East Asian culture where group harmony and social conformity are prioritized, adolescents may avoid direct confrontation to preserve the peace (130). Support-seeking within the family or peer group may be preferred. There may also be a tendency to suppress emotions and manage through emotional coping strategies (131).

*Problematic internet use (PIU)* – While PIU has been found to be associated with bully victimization in both East Asian and Western cultures, there are significant differences in the rates of PIU being much higher in East Asian countries and up to three times that in Western countries (132) eg 14% in China Vs 4% in the US (133). The higher rates of PIU may relate to adolescents gaining relief from academic pressure (134), a notable stressor in East Asian countries (135) or coping with anxiety (118, 119). Greater PIU in turn is associated with higher likelihood of bully victimization.

### 4.3 Study limitations

While most of the East Asian countries hold to collectivistic practices, a comparison of the degree to which it still informs policy and practice in each of the countries was not done. Within the Chinese diaspora, it was difficult to make comparisons due to the diversity across rural and urban areas and large variations in data found. The quality of papers varied significantly (**Table 2**) though the higher quality NOS papers were emphasized in synthesis. The time period for bullying victimization was inconsistent, with some studies reporting 30 days to lifetime versus the previous 12 months. It is possible that some evidence was omitted since non-English papers, published in Mandarin Chinese, Japanese, and Korean native languages were excluded limiting the comprehensiveness of the findings. In addition, the protocol for this scoping review has not been pre-registered. The intention was to pre-register the scoping review protocol on an open platform such as PROSPERO; however, PROSPERO does not accept scoping reviews.

### 4.4 Future research

The high prevalence of bullying among adolescent children suggests that more needs to be done to recognize and address the issue. The scoping review showed that a significant proportion of bullying occurred in schools through peer or teacher victimization. The whole school intervention approach (anti-bullying framework) (136–138) at four different levels of the individual, classroom, school and community has been shown to be very effective in reducing bullying victimization and increasing student satisfaction in Sweden. This approach could be the next steps of a further research initiative. The first step of applying the intervention would be to understand what is now occurring at these four levels in East Asian schools. A survey could be conducted to understand how adolescent students at an ‘individual level’ are getting help when victimization occurs, and their knowledge, attitudes and beliefs about asking for support.

### 4.5 Conclusion

Bullying prevalence rates varied across East Asia, from 6.1 – 61.3% in traditional bullying victimization and 3.3 – 74.6% in cyberbullying victimization, with higher prevalence rates seen in at-risk populations. Bullying correlated strongly with depression. Findings of this review suggest that risk and protective factors for bullying and victimization in East Asian cultures are very similar to those reported in Western cultures. The evidence suggests that strong relationships within families, peers and the school community coupled with adolescents’ positive coping style and high self-esteem are protective against the negative effects of bullying. Similar to Western cultures, adolescents who are bully-victims and poly-victims are most vulnerable to depression. Unique findings specific to East Asian culture are that adolescents who are perceived as ‘being different’ i.e., sexual minority, LBC are more likely to be bully victims and to experience depression. In East Asian cultures, teachers and parents who are figures of authority, may paradoxically be perpetrators of bullying and harsh physical punishment. Understanding bullying patterns, including purpose (for example, when the physical punishment is not for personal reasons), in East Asian cultures and systems of support in schools may offer further clues to providing support to bullying victims.

## Data Availability

The original contributions presented in the study are included in the article/**Supplementary Material**. Further inquiries can be directed to the corresponding author.
