# Supplementary material for: Bullying and depression among adolescents in East Asia: a scoping review on prevalence rates, risk and protective factors
Source: Front Psychiatry. 2025 Mar 5;16:1497866. doi: 10.3389/fpsyt.2025.1497866 (PMC11932047; doi:10.3389/fpsyt.2025.1497866)
Supplement: Supplementary file 1 [file Table1.docx]

Supplementary Material

# Supplementary Table

**Supplementary Table 1.** Definitions and descriptions of bullying and cyberbullying.

| **Terms** | **Definition and Description** |
| --- | --- |
| **Bullying** | The definition of bullying and the evaluation methods can be very variable. ‘Bullying is defined as being an aggressive, unwanted negative action that is carried out by one person or a group of people, repeatedly and over time against a victim who has difficulty defending themselves i.e., with power imbalance’ [1]. It can take different forms. Traditional bullying can be direct - physical (hitting, pushing, things taken) or verbal / relational (name calling, negative comments), or indirect with social exclusion and spreading rumors. ‘Relational victimization’ is defined as direct or indirect peer maltreatment where manipulation of the interpersonal relationships as the main source of harm [2]. These may include experiences of being ignored, excluded by peers, or being a target of malicious rumors [3]. |
| **Cyberbullying** | ‘Cyberbullying’ is the use of electronic media to engage in bullying acts while ‘cyber victims’ are individuals who have experienced bullying through these media. Cyberbullying can be harder to define because a single act of bullying can be passed on many times by others (and does not necessarily fulfil criteria for a repeated act [4]. |
| **Victim, Bully (i.e., perpetrator), Bully-victim, and Polyvictimization** | In bullying literature, there are three different groups described including the ‘victim’, the ‘bully’ or ‘perpetrator’ and the ‘bully-victim’ or ‘victim-perpetrator’. ‘Victims’ are individuals who have experienced bullying, while ‘bullies’ or ‘perpetrators’ are persons who have carried out acts of bullying. The ‘bully-victim’ is a person who has both been a victim and who perpetuates acts of bullying [1]. The ‘bully-victim’, unlike the ‘bully only’ or ‘victim only’ group is most vulnerable with much poorer educational and socio-emotional outcomes. They are more likely to be anxious, over aroused [5] with impulse control problems, be socially isolated, have difficulties making friends [5, 6], and to do poorly academically [7]. Polyvictimization’ refers to having been the victim of multiple forms of victimization i.e., physical, sexual, peer, neighborhood violence [8]. |

**References**

1. Lim LL. The influences of harmony motives and implicit beliefs on conflict styles of the collectivist. *International Journal of Psychology* 2009;44(6):401-9.
2. Xu W, Huang Y, Tang W, Kaufman MR. Heterosexual marital intention: The influences of Confucianism and stigma among Chinese sexual minority women and men. *Archives of Sexual Behavior* 2022;51(7):3529-40.
3. Vivolo-Kantor AM, Martell BN, Holland KM, Westby R. A systematic review and content analysis of bullying and cyber-bullying measurement strategies. *Aggress Violent Behav.* 2014;19(4):423-434. doi:10.1016/j.avb.2014.06.008.
4. Lazzara E. ‘Our Lives from a Different Perspective’: How Chinese and Taiwanese Gay and Lesbian Individuals and Their Parents Navigate Confucian Beliefs. In *Intersecting Religion and Sexuality* 2020 Oct 5 (pp. 190-211). Brill.
5. Liao X, Jia Y, Yang Y, Zhang W. Association between bullying victimization and mental health problems among Chinese left-behind children: A cross-sectional study from the Adolescence Mental Health Promotion Cohort. *Frontiers in Psychiatry* 2024;15:1440821.
6. Juvonen J, Graham S, Schuster MA. Bullying among young adolescents: The strong, the weak, and the troubled. *Pediatrics* 2003;112(6):1231-7.
7. Wu, W., Qu, G., Wang, L., Tang, X., & Sun, Y. H. (2019). Meta‐analysis of the mental health status of left‐behind children in China. *Journal of paediatrics and child health*, *55*(3), 260-270.
8. Kim DH, Kim KI, Park YC, Zhang LD, Lu MK, Li D. Children's experience of violence in China and Korea: a transcultural study. *Child Abuse Negl*. 2000;24(9):1163-1173. doi:10.1016/s0145-2134(00)00175-7
